# A comprehensive experimental and theoretical perspective of novel triazole-based pyridine and quinoline derivatives for corrosion protection of carbon steel in sulfuric acid solution

**DOI:** 10.1038/s41598-025-10619-5

**Published:** 2025-07-24

**Authors:** Kamelia Belal, A. H. El-Askalany, Eslam A. Ghaith, Ahmed Fathi Salem Molouk

**Affiliations:** 1 Department of Chemistry, Faculty of Science, Mansoura University, Mansoura, 35516 Egypt; 2 Faculty of Science, Mansoura University Sustainable Energy Research Lab (MSER), Mansoura University, Mansoura, 35516 Egypt; 3 Faculty of Science, New Mansoura University, New Mansoura City, Egypt

**Keywords:** Corrosion, Chemical engineering, Corrosion

## Abstract

Herein, we synthesized two novel triazole compounds (KB1 and KB2). Their efficiency in inhibiting corrosion of carbon steel (CS) in 0.5 M H_2_SO_4_ was evaluated using open circuit potential (OCP) vs. time, potentiodynamic polarization (PP), electrochemical impedance spectroscopy (EIS), surface characterization methods, and computational studies. Based on EIS, the corrosion protection capacities of KB1 and KB2 increased with increasing concentration, reaching 86.9% and 92.4% at 9 × 10^− 5^ M at 298 K for KB1 and KB2, respectively, which are consistent with the findings obtained *via* PP. The PP curves imply that KB1 and KB2 act as mixed-type inhibitors. Meanwhile, their adsorption on the surface of CS obeyed the Langmuir isotherm. Using EIS and PP approaches, the effect of temperature on corrosion behavior was examined. As the temperature increased from 298 to 318 K, the efficacy of the inhibitors improved, then decreased at 328 K. This suggests that KB1 and KB2 may desorb from the CS at 328 K. The activation and adsorption parameters were computed and discussed. The effect of immersion time on CS corrosion was documented. The *R*_*ct*_ values of the KB2 compound peaked at 2770.0 Ω cm^2^ after 6 h of immersion, later declining to 2685.0 Ω cm^2^ after 93 h, thus evidencing the enhanced stability of the inhibitor’s protective layer on the CS surface. Additionally, the steel surface was found to be positively charged in the H_2_SO_4_ solution, as indicated by the potential of zero charge (PZC) measurements using EIS. Surface morphology analysis using Atomic force microscopy (AFM), X-ray photoelectron spectroscopy (XPS), and Fourier transform infrared spectroscopy (FT-IR) demonstrated the formation of a protective layer with a high degree of coverage at 9 × 10^− 5^ M. The test solutions were analyzed *via* UV-visible spectroscopy to determine whether complex formation between inhibitor molecules and Fe^2+^ ions is possible. The mechanism of inhibition was better understood *via* quantum chemical indices (based on DFT). The configurational adsorption performance of KB1 and KB2 on the Fe (110) surface was investigated *via* Monte Carlo (MC) simulation, which revealed that inhibitors adsorbed on the Fe surface in a flat orientation. These results are thought to have some bearing on the sensible development of potent inhibitors for acidic corrosion.

**Supplementary Information:**

The online version contains supplementary material available at 10.1038/s41598-025-10619-5.

## Introduction

Carbon steel (CS) is the primary material used in construction and structural applications for producing various industrial tools, including those for oil and water pipelines, power plants, etc^1^. It is valued for its high strength, widespread availability, affordability, and ease of welding. However, despite these benefits, CS is prone to corrosion when subjected to aggressive solutions, causing significant financial losses^2^. Sulfuric acid is a strong oxidizer and a dehydrator. It interacts violently with water and can ignite other materials. It is, therefore, categorized as a hazardous material, requiring safe transportation and storage to avoid any mishaps^3^. It is the most used acidic solution in industrial applications to eliminate oxides produced by chemical processes in steel and oil refineries. These acid solutions can harm metals used to build storage tanks and pipelines as time passes^4^. One of the most popular and extensively used corrosion control strategies in a wide range of industries is the implementation of corrosion inhibitors, which are a flexible and effective method of reducing corrosion^5–12^. Corrosion inhibitors are organic or inorganic compounds introduced in trace amounts to a corrosive medium and impede the anodic and/or cathodic metal corrosion reactions^13,14^. Due to their high adsorption efficiency, environmental friendliness, and adjustable chemical structures, organic corrosion inhibitors have garnered increasing interest in recent years. Thanks to developments in molecular design, inhibitors with heteroatoms (N, O, and S) and numerous adsorption sites may now be synthesized, improving their capacity to create durable protective films on metal surfaces. Adsorption can occur chemically (chemisorption), physically (physisorption), or a combination of both^15^. Recent research has confirmed the adaptability of organic inhibitors in practical applications by demonstrating their efficiency under various corrosive conditions^16–28^. These results are consistent with the current study’s approach and encourage further investigation of novel hybrid inhibitors with enhanced adsorption capacities and sustainability profiles. Significantly, nitrogen-containing heterocyclic compounds, like triazole derivatives, have been widely researched for their chemical and biological characteristics and are commonly utilized in medicinal chemistry. Apart from their biological roles, they are acknowledged for their potency in impeding corrosion in acidic conditions^29–34^. Triazoles are five-membered heterocyclic compounds containing three nitrogen atoms with lone pairs and π-electrons, which are strongly adsorbed on metal surfaces. Nearby heteroatoms (N–N) improve adsorption on the metal surfaces^35^. Additionally, their application in the pharmaceutical sector demonstrated their non-hazardous qualities^36^. Based on previous studies, compounds containing pyridine and quinoline have strong corrosion-inhibiting properties^37–42^. The significance of choosing the tested inhibitors as corrosion inhibitors lies in their economic feasibility and the presence of an electron-rich system, such as an aromatic ring and heteroatoms^43,44^. By establishing coordination bonds with the metal surface, these atoms can produce a protective layer that lessens corrosion. Moreover, they can be used in practical applications because their synthesis is economical and environmentally friendly. Consequently, the innovation of selecting these inhibitors is the cost-effective molecular design that combines three extremely potent moieties into one inhibitor molecule: hydrazinyl triazole, clubbed pyridine/ quinoline. These moieties are well-known for their potent electron-donating capabilities and potential to create coordination bonds with metal surfaces. Combining them results in a structure with more adsorption sites, higher electron density, and better surface contact, all boosting corrosion inhibition performance. Moreover, the compounds were synthesized *via* an environmentally friendly ultrasound-assisted method. Since corrosion inhibition literature has not yet documented this design in conjunction with an ultrasound-assisted green synthesis method, it provides a novel and sustainable approach. As a result, we assessed the ability of (*Z*)-4-amino-5-(2-(pyridin-4-ylmethylene)hydrazinyl)-4*H*-1,2,4-triazole-3-thiol (KB1) and (*Z*)-4-amino-5-(2-((2-azidoquinolin-3-yl)methylene)hydrazinyl)-4*H*-1,2,4-triazole-3-thiol (KB2) to impede the dissolution of CS in 0.5 M H_2_SO_4_ utilizing electrochemical techniques. The CS’s surface morphology, composition, and structure were determined using various characterization techniques: AFM, XPS, and FT-IR. The test solutions were analyzed *via* UV-Visible spectroscopy to check the complex formed among ferrous ions and the investigated inhibitors. Additionally, computational studies *via* quantum calculations (DFT) and Monte Carlo (MC) simulations are conducted to reinforce the experimental outcomes.

## Experimental techniques

### Working electrode preparation

The utilized CS sheet has the following compositions (weight%): C 0.2%, Si 0.25%, S 0.05%, Mn 0.5%, and Fe 99%. It was sliced into 1 cm x 1 cm pieces and firmly cemented in an epoxy/hardener mixture. This guaranteed that the coupon used in the electrochemical measurements would have an exposed area of 1 cm². Before testing, the working electrode was polished using a series of emery paper grades (320, 400, 1000, and 2000) to achieve a mirror surface, cleaned with double-distilled water, and dehydrated with filter papers.

### General procedure for the synthesis of inhibitors

**Fig. 1:**
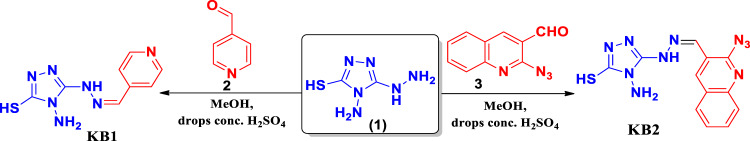
Synthesis of inhibitors.

As indicated in Fig. 1, a mixture of 4-amino-5-hydrazineyl-4*H*-1,2,4-triazole-3-thiol (**1**) (0.146 g,1 mmol) and pyridine-4-carboxaldehyde (**2**) (0.094 mL, 1 mmol) or 2-azidoquinoline-3-carbaldehyde (**3**) (0.198 g, 1 mmol) was mixed and irradiated under ultrasound for 3 min at ambient temperature in MeOH (20 mL) containing 3 drops of conc. H_2_SO_4_ as a catalyst. The formed precipitates (KB1 and KB2) were filtered off and washed with hot MeOH. Figures S1–S6 in the supplementary file display the ^1^HNMR, ^13^CNMR, and mass spectra, while Table S1 presents further details of the synthesis process of the inhibitors.

(*Z*)-4-amino-5-(2-(pyridin-4-ylmethylene)hydrazinyl)-4*H*-1,2,4-triazole-3-thiol (KB1). Yield, 92%; orange powder; m.p 244–245 °C. IR (ν_max_, cm^− 1^): 3439 (NH_2_), 3266 (NH), 3084 (sp^2^ CH), 1622, 1618, 1577, 1557 (C = N & C = C). ^1^HNMR (DMSO-*d*_6_, 500 M*Hz*): *δ* (ppm) 5.57 (s, 2 H, NH_2_), 7.84 (d, *J* = 6.5 *Hz*, 2 H), 8.34 (s, 1 H, CH), 8.69 (d, *J* = 6 *Hz*, 2 H), 11.51 (s, 1 H, NH), 13.15 (s, 1 H, SH). ^13^CNMR (DMSO-*d*_*6*_, 125 M*Hz*): *δ* (ppm) 121.62 (2 C), 139.38, 146.08 (2 C), 146.43, 149.00, 164.99. )EMIS) m/z (%): 235.71 (M^+^, 44.25%), 191.80 (90.74%), 145.14 (100%, base peak), 114.83 (63.39%), 104.17 (63.56%), 95.92 (55. 72%), 94.70 (89.00%), 62.30 (58.22%).

(*Z*)-4-amino-5-(2-((2-azidoquinolin-3-yl)methylene)hydrazinyl)-4*H*-1,2,4-triazole-3-thiol (KB2). Yield, 91%; brown powder; m.p 238–239 °C. IR (ν_max_, cm^− 1^): 3415 (NH_2_), 3292 (NH), 3035) sp^2^ CH), 1674, 1641, 1614, 1547 (C = N & C = C). ^1^HNMR (DMSO-*d*_6_, 400 M*Hz*): *δ* (ppm) 3.55–3.67 (br, 2 H, NH_2_), 7.82 (t, *J* = 7.4 *Hz*, 1 H), 7.97 (t, *J* = 7.8 *Hz*, 1 H), 8.36 (d, *J* = 8 *Hz*, 1 H), 8.57 (s, 1 H, CH), 8.61 (d, *J* = 8.4 *Hz*, 1 H), 8.93 (s, 1 H, CH), 11.48 (s, 1 H, NH), 13.16 (s, 1 H, SH). ^13^CNMR (DMSO-*d*_*6*_, 100 M*Hz*): *δ* (ppm) 116.65, 120.51, 124.47, 127.84, 128.84, 130.16, 130.55, 131.92, 136.67, 146.76, 149.62, 165.11. )EMIS) m/z (%): 326.69 (M^+^, 23.26%), 242.34 (100%, base peak), 211.52 (67.21%), 206.05 (63.55%), 127.03 (96.45%), 121.16 (81.45%), 109.90 (68.40%), 76.99 (92.30%).

### Solutions

The corrosive medium, 0.5 M H_2_SO_4_, was prepared by diluting the AR-grade sulfuric acid solution with double-distilled water. Economic and practical considerations were the key factors in selecting the tested inhibitors’ concentrations. It would be ineffective to use concentration over the saturation point, and doing so might result in needless expenses and possible environmental issues. To ensure efficient corrosion prevention while maintaining cost-effectiveness and minimizing excessive chemical use, the tested concentrations were selected. Therefore, stock solutions of the tested inhibitors (10^− 3^ M) were prepared. Dimethyl sulfoxide (10 mL) was used to dissolve the calculated amount of inhibitor, and it was then brought to 100 mL with absolute ethanol. Different concentrations of the investigated inhibitors (1–9 × 10^− 5^ M) were employed by dilution from the stock solution. The percentage of solvents in which the inhibitor dissolved was maintained throughout the prepared solutions, both with and without the inhibitors, to prevent the effect of solvents on the inhibition. No precipitation or turbidity was observed throughout the experiments, indicating good solubility and stability of the inhibitors in the test solutions.

### Electrochemical measurements

Electrochemical tests were carried out in a corrosion cell using three electrodes: a CS working electrode, a platinum wire as the counter electrode, and a saturated calomel electrode (SCE) as the reference electrode. The three electrodes were immersed in the test solutions for 35 min before each experiment to achieve a stabilized OCP. EIS measurements were performed using AC signals of 10 mV amplitude at OCP and the frequency range from 100 k*Hz* to 0.1 *Hz*. The PP measurements were achieved by sweeping the potential from − 500 mV to 500 mV against OCP using a scan rate of 0.5 mV/s. All experiments were performed with newly prepared solutions at 298 K *via* a Potentiostat/Galvanostatic/ZRA analyzer (Gamry 5000E, USA). At each inhibitor concentration, the experiments were repeated three times to ensure reproducibility and accuracy of the corrosion inhibition results.

### Zero charge potential

EIS was implemented to estimate the potential of zero charge (PZC). It was carried out at various potentials with an AC amplitude of 10 mV in 0.5 M H_2_SO_4_ solutions with 5 × 10^− 5^ M of KB1 and KB2. A plot has been created to show how the applied voltage affects the variation of C_dl_. E_PZC_ represents the lowest C_dl_ value.

### Surface characterization

CS specimens were polished with different grades of emery papers, washed, dried, and exposed to 0.5 M H_2_SO_4_ solution without and with adding 9 × 10^− 5^ M of KB1 and KB2 for 24 h, after that the specimens were brought out and dried to analyze the morphology *via* Nanosurf FlexAFM 3, Gräubernstrasse 12, 4410 Liestal, Switzerland for AFM analysis and the components of the film that developed on the CS was investigated *via* AXIX Ultra DLD, Kratos, UK for XPS analysis. FTIR analysis detects the functional groups present in the inhibitor and provides qualitative insight into the inhibitor’s adsorption onto the CS surface.

### UV–Visible spectra

Corrosion protection was examined by UV-Visible spectrophotometry both before and after the CS coupons were submerged for 48 h to gather additional information on the complex formed between Fe^2+^ ions and the tested inhibitors. The spectra were recorded *via* a T80 + UV/vis spectrometer (UK).

### Theoretical computations

The energy minimization of KB1 and KB2 in an aqueous medium was investigated through DFT calculations using the GGA method with BOP functional and the DNP basis set in the Dmol^3^ module of BIOVIA Materials Studio 2017 software^45,46^. Based on DFT calculations, several quantum indices were calculated^47^. The Adsorption Locator module was also applied for the MC simulation^48^. Following the use of the COMPASS force field to optimize the geometry of both the tested molecules and Fe (1 1 0), the Adsorption Locator module uses Monte Carlo searches to determine the feasible adsorption configurations of the inhibitors on the surface and their influence on the inhibitory performance^49^.

## Results and discussion

### Open circuit potential (OCP)

**Fig. 2:**
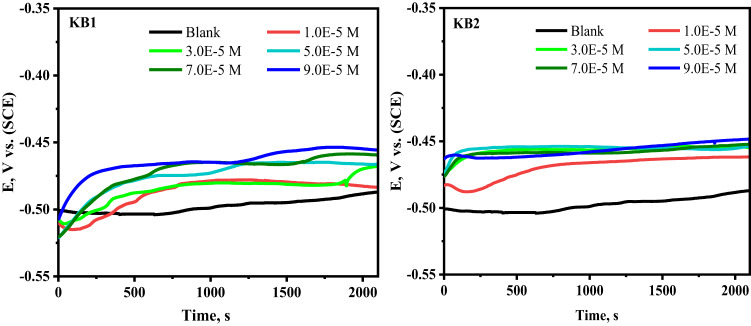
OCP-time curves for CS submerged in 0.5 M H_2_SO_4_ before and after varying doses of KB1 and KB2 at 298 K.

The steady-state potential was measured by recording the change in OCP values after 35 min of exposure to 0.5 M H_2_SO_4_, both without and with inhibitors at various concentrations (Fig. 2). After 10 min, the OCP curves become nearly linear. The presence of KB1 and KB2 caused the OCP curves to stabilize at more positive values, indicating the formation of a protective layer on the CS surface, primarily on the anodic sites^50,51^.

### PP analysis

**Fig. 3:**
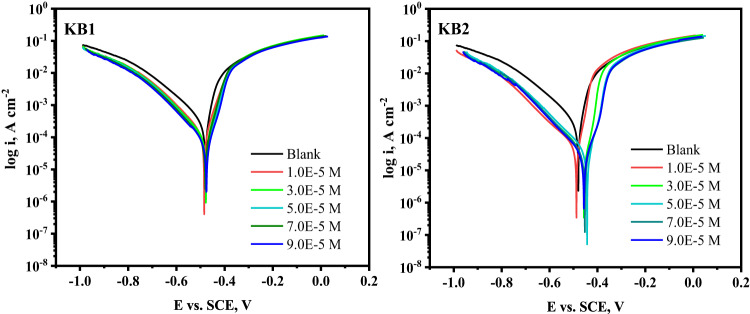
Tafel diagrams for CS in 0.5 M H_2_SO_4_, both without and with KB1 and KB2 at 298 K.

Tafel polarization graphs for CS in 0.5 M H_2_SO_4_ at 298 K without and with different concentrations of KB1 and KB2 are displayed in Fig. 3. Table 1 provides the electrochemical parameters obtained from Tafel plots. Equation (1) was applied to find the inhibition effectiveness based on the *i*_*corr*_ values^52^.

1 $$\:\% \:{\text{IE = }}\:\theta  \times \:\:{\text{100}}\:{\text{ = }}\:\left( {{\text{1 - }}\frac{{\:{i_{corr}}}}{{{i^\circ }{\:_{corr}}}}} \right) \times \:{\text{100}}$$.

*i*$\:^\circ\:$_*corr*_ and *i*_*corr*_ correspond to the corrosion current densities before and after the addition of the inhibitor. It is evident from Table 1 that *i*_*corr*_ declines as the inhibitor’s concentration rises, reaching the lowest corrosion rate value at 9 × 10^− 5^ M. This phenomenon can be explained by the inhibitors adhering to the CS surface and establishing a protecting layer that shields the surface from the harsh environment, enhancing inhibition capacity values, which reached 87.5% and 91.4% for KB1 and KB2, respectively^53^. The Tafel slopes (*β*_*a*_, *β*_*c*_) do not alter with varying doses of KB1 and KB2, suggesting that their addition has no effect on the corrosion mechanism and that the principal mechanism of inhibition is the adsorption of inhibitors onto the CS surface, which blocks its active sites^54^. As seen from the E_corr_ values in Table 1, there is a shift (< 85 mV) from the E_corr_ of the blank when inhibitor molecules are present. This implies that the substances being studied have mixed-type inhibitory effects^55^. Additionally, using Eq. (2), the polarization resistances (*R*_*p*_) for the CS/H_2_SO_4_ system at various concentrations of KB1 and KB2 were determined:

2 $$\:{R}_{p}\:=\:\frac{\beta\:a\beta\:c}{{2.303(\beta\:a+\beta\:c)i}_{\text{c}\text{o}\text{r}\text{r}}}$$.

As the concentration rose, the R_p_ dramatically increased, indicating the formation of a protective film of KB1 and KB2 on the CS surface^56^.

**Table 1:** Corrosion parameters based on extrapolation of CS Tafel curves at 298 K.

Inhibitors	Conc. (M)	-E_corr_, mV vs. SCE	i_corr_. µA cm^− 2^± SD	β_a_, mVdec^− 1^	β_c_, mVdec^− 1^	C.*R*., mpy	*R*_*p*_, Ω cm²	θ	%IE
Blank	0.5 M H_2_SO_4_	480.0	327.0 ± 0.16	53.80	159.3	149.5	53.40	–	–
KB1	1 × 10^− 5^	483.0	120.0 ± 0.29	49.70	112.4	54.91	129.70	0.633	63.3
	3 × 10^− 5^	478.0	93.20 ± 0.16	44.60	110.7	42.56	148.12	0.715	71.5
	5 × 10^− 5^	467.0	82.10 ± 0.07	48.40	133.5	37.48	187.87	0.749	74.9
	7 × 10^− 5^	473.0	52.00 ± 0.15	38.00	66.80	23.76	202.26	0.841	84.1
	9 × 10^− 5^	470.0	41.00 ± 0.04	41.70	63.60	18.73	266.74	0.875	87.5
KB2	1 × 10^− 5^	457.0	71.00 ± 0.73	75.00	155.8	32.43	309.63	0.783	78.3
	3 × 10^− 5^	445.0	39.40 ± 0.41	42.80	141.7	17.98	362.27	0.880	88.0
	5 × 10^− 5^	452.0	31.80 ± 0.49	39.90	132.6	14.53	418.80	0.903	90.3
	7 × 10^− 5^	459.0	29.30 ± 0.34	47.80	131.7	13.38	519.74	0.910	91.0
	9 × 10^− 5^	458.0	28.20 ± 0.57	49.70	133.5	12.87	557.66	0.914	91.4

### EIS measurements

**Fig. 4:**
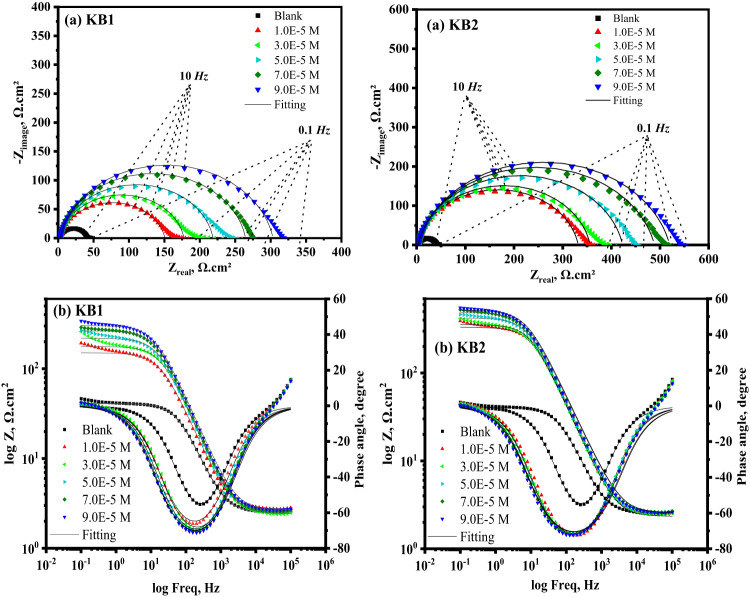
Nyquist (**a**) and Bode (**b**) plots for CS in 0.5 M H_2_SO_4_ without and with various doses of KB1 and KB2 at 298 K.

**Fig. 5:**
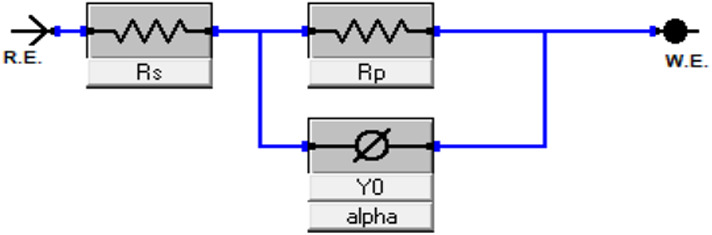
The applied equivalent electrical circuit for fitting EIS data.

EIS, a non-destructive technique, was used to investigate the interface of CS substrates with and without inhibitors. It provides valuable insights into the corrosion inhibition mechanisms by revealing the electro-resistive and capacitive properties of the substrates under investigation^57^. The Nyquist and Bode diagrams of CS, produced in 0.5 M H_2_SO_4_ solution with and without different doses of KB1 and KB2 at 298 K, are displayed in Fig. 4a and b, respectively. As observed in Fig. 5, the acquired impedance values were fitted to a standard electrochemical circuit. Table 2 provides a summary of the evaluated corrosion parameters. Equation (3) can be utilized to get % IE based on the charge-transfer resistance (*R*_*ct*_) values^58^:

3 $$\:\% \:{\text{IE = }}\:\theta  \times \:\:{\text{100}}\:{\text{ = }}\:\left( {{\text{1 - }}\frac{{{R^\circ }{\:_{ct}}}}{{{R_{ct}}}}} \right) \times \:{\text{100}}$$.

where “*R*^*o*^_*ct*_” represents the charge-transfer resistance in blank solution and “*R*_*ct*_” represents the charge-transfer resistance using different doses of KB1 and KB2. Nyquist plots (Fig. 4a) clearly show that the spectra consist of a single capacitive semicircle, indicating that the charge transfer process primarily controls the dissolution of CS, and the adsorption of KB1 and KB2 is achieved by simple surface coverage. As opposed to the uninhibited solution, the capacitive semicircle size of CS significantly increased upon the addition of the different concentrations of KB1 and KB2, implying a decrease in the rate of CS corrosion and an increase in % IE^59^. This means that the surface in the aggressive solution is protected *via* the developed adsorbed layer. The Bode graphs for KB1 and KB2 are displayed in Fig. 4b. Upon elevation of the inhibitor concentration, the impedance value rose; the higher impedance for KB2 suggests that it provides more safeguards for CS than KB1^34^. Additionally, Nyquist plots deviate from idealized semicircles. This depression of the semicircles is attributed to many factors, including frequency dispersion effects, electrode surface irregularities, grain boundary impedance, and impurities^60^. The values of *R*_*ct*_ (Table 2) increase from 39.50 Ω.cm^2^ for uninhibited CS to 300.7 Ω.cm^2^ and 517.4 Ω.cm^2^ at 9 × 10^− 5^ M of KB1 and KB2, respectively. The formation of thin layers of inhibitors at the CS-corrosive solution interface caused this observation^61^. As the concentrations of KB1 and KB2 rise, the double-layer capacitance (C_dl_) values decrease, signifying that KB1 and KB2 molecules, which have lower dielectric constants, displace water molecules on the CS^62^. Analyzing C_dl_ data *via* the constant phase element (CPE) data parameters ($\:{\text{Y}}_{0}$ and n) was achieved, whereas the CPE parameters for the circuit displayed in Fig. 5 were utilized to calculate C_dl_ *via* the following formula^63^:

4 $$\:\:{\text{C}}_{\text{dl}}\:={\left({\text{Y}}_{0}\:{R}_{ct}^{\left(1-\text{n}\right)}\right)}^{\raisebox{1ex}{$1$}\!\left/\:\!\raisebox{-1ex}{$\text{n}$}\right.}$$.

where the CPE exponent n, depends on the character of the CS surface, while Y_0_ is the CPE magnitude^64^. The data collected using the PP approach is supported by the EIS findings.

**Table 2:** The parameters obtained from EIS for KB1 and KB2 at 298 K.

Inhibitors	Conc. (M)	*R*_s,_ Ω cm²	*R*_ct,_ Ω cm²± SD	Y_o_ x10^− 6^, µΩ^−1^s^*n*^cm^−2^	*n*	C_dl,_ µF.cm^− 2^	Goodness of fit (x10^− 3^)	θ	%IE
Blank	0.5 M H_2_SO_4_	2.520	39.50 ± 0.24	125.1	0.907	72.4	3.732	--	--
KB1	1 × 10^− 5^	2.744	148.0 ± 0.82	100.4	0.883	57.6	5.432	0.733	73.3
	3 × 10^− 5^	2.423	175.2 ± 0.41	75.36	0.900	46.6	8.130	0.775	77.5
	5 × 10^− 5^	2.630	216.0 ± 0.16	72.19	0.902	46.0	4.991	0.817	81.7
	7 × 10^− 5^	2.563	262.0 ± 0.33	68.16	0.900	43.6	4.089	0.849	84.9
	9 × 10^− 5^	2.660	300.7 ± 0.41	66.25	0.891	40.9	4.109	0.869	86.9
KB2	1 × 10^− 5^	2.477	329.5 ± 0.53	70.81	0.900	46.7	4.828	0.880	88.0
	3 × 10^− 5^	2.333	354.0 ± 0.61	68.93	0.901	45.7	4.535	0.888	88.8
	5 × 10^− 5^	2.543	420.6 ± 0.78	68.80	0.891	44.5	3.500	0.906	90.6
	7 × 10^− 5^	2.574	486.1 ± 0.65	67.93	0.870	40.8	3.406	0.919	91.9
	9 × 10^− 5^	2.541	517.4 ± 0.57	65.20	0.871	39.4	3.341	0.924	92.4

### Effect of temperature

**Fig. 6:**
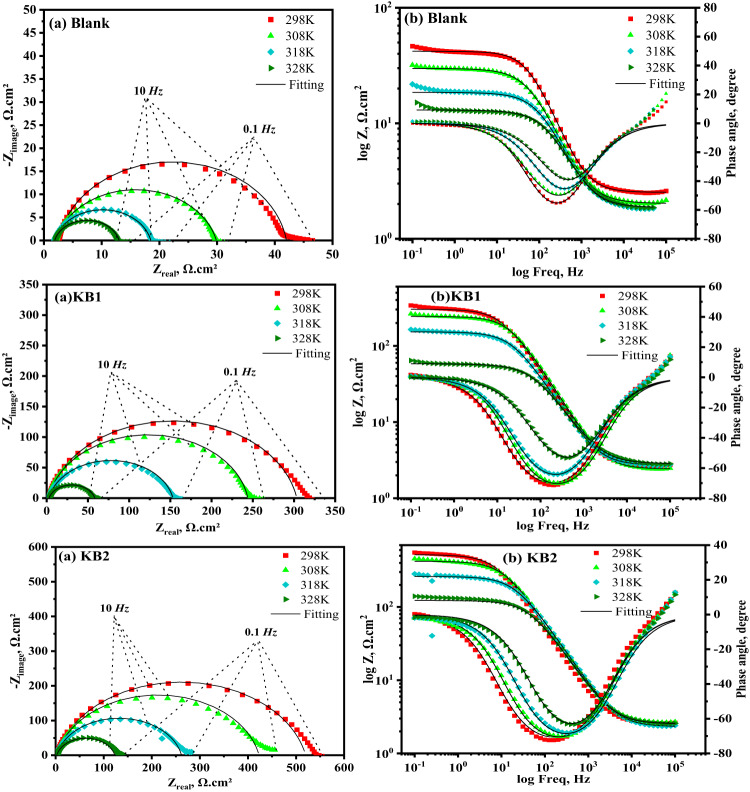
Nyquist (**a**) and Bode (**b**) curves for CS in 0.5 M H_2_SO_4_ without and with adding 9 × 10^− 5^ M of KB1 and KB2 at different temperatures.

**Fig. 7:**
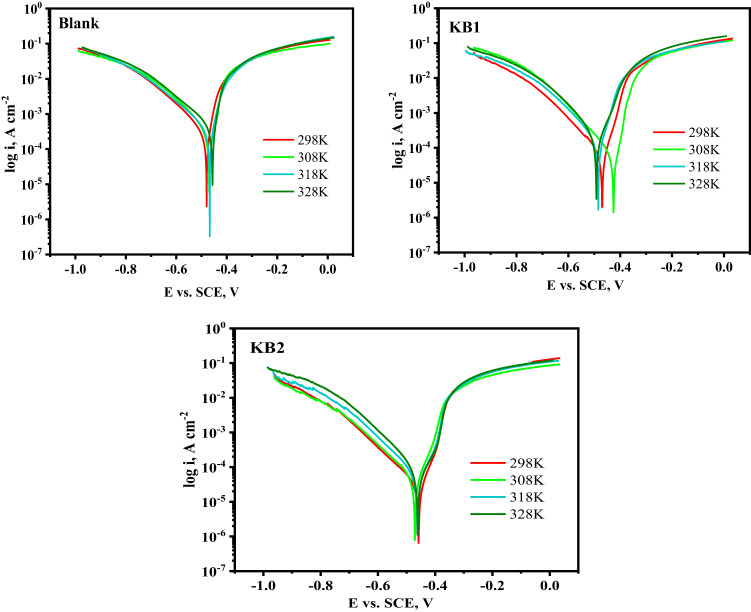
PP curves for CS in 0.5 M H_2_SO_4_ without and with adding 9 × 10^− 5^ M of KB1 and KB2 at different temperatures.

**Fig. 8:**
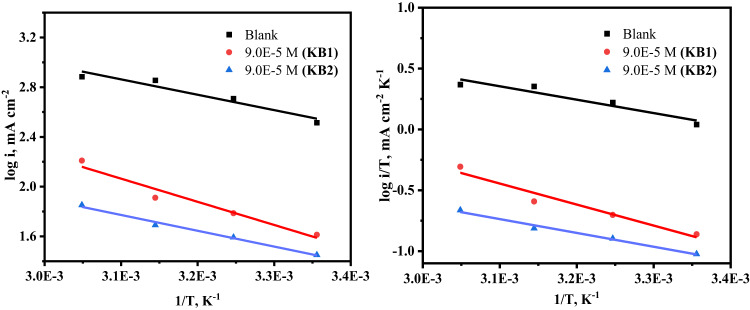
Arrhenius (left) and transition state (right) plots for CS in 0.5 M H_2_SO_4_ without and with 9 × 10^− 5^ M of KB1 and KB2.

The inhibition potency of KB1 and KB2 for the dissolution of CS in 0.5 M H_2_SO_4_ in the absence and presence of the maximum concentration was evaluated at various temperature ranges (298–328 K) *via* EIS and PP measurements. Increased system temperature enhances the kinetic mobility of corrosive species. By decreasing the deposited corrosion products and even the adsorbed film covering the CS surface, this behavior exposes a larger area of the CS to the corrosive solution, which in turn enhances iron oxidation and H_2_ evolution^65^. As revealed in Fig. 6a, the Nyquist spectra of CS in 0.5 M H_2_SO_4_ without and with the addition of 9 × 10^− 5^ M of KB1 and KB2 showed a semicircular shape that shrank in diameter as the temperature rose, suggesting that the mechanism underlying the CS corrosion process is independent of temperature. As the temperature rose, the data of % IE in Table 3 increased marginally, reaching 88.6% and 93.2% for KB1 and KB2, respectively. This demonstrated their capacity to inhibit and adsorb onto the surface of CS as a stable film over a range of temperatures, protecting it from the damaging effects of 0.5 M H_2_SO_4_ solution *via* chemical bonds (chemical adsorption)^66^. At 328 K, the % IE values decreased, which indicates that higher temperatures (328 K) may have led to the desorption of KB1 and KB2 from the surface. The temperature effect caused a slight increase in corrosion. The Bode diagrams in Fig. 6b show a one-time constant at all temperatures, confirming the presence of a single capacitive loop. Increasing temperature decreases the impedance modulus values at lower frequencies for the systems under study (Fig. 6b)^64^. PP measurements revealed similar findings, which are shown in Fig. 7 and Table 4. As temperature rises, so does the *i*_*corr*_ and the rate at which CS dissolves. Since the i_corr_ values obtained in the existence of KB1 and KB2 were lower than those achieved in their absence, it is evident that the investigated inhibitors significantly hindered the corrosion response of CS. The activation energy ($\:{\text{E}}_{\text{a}}^{\text{*}}$), change in enthalpy, and entropy (ΔH^∗^, ΔS^∗^) presented in Table 5 are thermodynamic activation parameters of the CS corrosion computed based on PP utilizing the Arrhenius and transition state equations as follows^67^.

5 $$\:\text{log}{\text{i}}_{\text{c}\text{o}\text{r}\text{r}}=\:-\frac{{\text{E}}_{\text{a}}^{\text{*}}}{2.303\text{R}\text{T}}+\text{l}\text{o}\text{g}\left(\text{A}\right)$$

6 $$\:\text{l}\text{o}\text{g}\left(\frac{{\text{i}}_{\text{c}\text{o}\text{r}\text{r}}}{\text{T}}\right)=\text{log}\left(\frac{\text{R}}{\text{N}\text{h}}\right)+\left(\frac{{\triangle\text{S}}^{\text{*}}}{2.303\text{R}}\right)-\left(\frac{{\triangle\text{H}}^{\text{*}}}{2.303\text{R}\text{T}}\right),$$.

where the Arrhenius pre-exponential factor is denoted by A, T denotes the absolute temperature(K), the universal gas constant corresponds to R (J K^− 1^ mol^− 1^), h stands for Planck’s constant, and N for the Avogadro number. The $\:{\text{E}}_{\text{a}}^{\text{*}}$ values were computed *via* the slope value of the Arrhenius plot in Fig. 8, as appeared in Table 5. ΔH^∗^ and ΔS^∗^ were computed *via* plotting log(*i*/T) against 1/T (Fig. 8), as indicated in Table 5. It was found that the values of $\:{\text{E}}_{\text{a}}^{\text{*}}$ are higher when the inhibitors are present than when they are not. When compared to the KB1 molecule, KB2 has a lower value of $\:{\text{E}}_{\text{a}}^{\text{*}}$, which indicates that its adsorption on the CS is chemical. However, the rise in the $\:{\text{E}}_{\text{a}}^{\text{*}}$ could not be considered significant because of competitive adsorption with water, whose desorption from the surface also requires some activation energy, as documented in literature^68^. The data gathered showed that the energy barrier for the dissolution reaction rises when inhibitors are present, and the dissolution becomes more difficult to happen with the existence of tested inhibitors, as implied by the positive values of ΔH*, which suggests that the dissolution reaction is an endothermic process^69^. The negative values of ΔS* in the existence of KB1 and KB2 suggest that the activated complex favors association over dissociation, indicating the establishment of a stable inhibitor layer on the CS surface^70^.

**Table 3:** The parameters obtained from EIS in 0.5 M H_2_SO_4_ before and after adding 9 × 10^− 5^ M of KB1 and KB2 at various temperatures.

Temp, K	Compound	*R*_s_, Ω cm²	*R*_ct_, Ω cm² ± SD	Goodness of fit (x10^− 3^)	θ	%IE
298	Blank	2.520	39.50 ± 0.24	3.732	–	–
	KB1	2.660	300.7 ± 0.41	4.109	0.869	86.9
	KB2	2.541	517.4 ± 0.57	3.341	0.924	92.4
308	Blank	2.018	27.86 ± 0.85	6.548	–	–
	KB1	2.428	241.5 ± 0.53	3.849	0.885	88.5
	KB2	2.609	420.3 ± 0.61	3.581	0.934	93.4
318	Blank	1.825	16.70 ± 0.57	2.737	–	–
	KB1	2.714	150.4 ± 0.41	3.447	0.889	88.9
	KB2	2.316	259.4 ± 0.69	5.030	0.936	93.6
328	Blank	1.862	11.17 ± 0.80	2.663	–	–
	KB1	2.841	55.46 ± 0.76	2.942	0.799	79.9
	KB2	2.532	120.4 ± 0.86	3.022	0.907	90.7

**Table 4:** The parameters obtained from PP in 0.5 M H_2_SO_4_ before and after adding 9 × 10^− 5^ M of KB1 and KB2 at various temperatures.

Temp, K	Compound	−E_corr_, mV vs. SCE	i_corr_, µAcm^− 2^ ± SD	C.*R*. mpy	θ	%IE
298	Blank	480.0	327.0 ± 0.16	149.5	–	–
	KB1	470.0	41.00 ± 0.04	18.73	0.875	87.5
	KB2	458.0	28.20 ± 0.57	12.87	0.914	91.4
308	Blank	454.0	511.0 ± 0.89	233.4	–	–
	KB1	457.0	61.10 ± 0.57	27.92	0.880	88.0
	KB2	475.0	39.10 ± 0.65	17.88	0.923	92.3
318	Blank	467.0	717.0 ± 0.82	327.7	–	–
	KB1	477.0	81.40 ± 0.61	37.19	0.886	88.6
	KB2	481.0	49.00 ± 0.89	22.39	0.932	93.2
328	Blank	460.0	766.0 ± 0.78	349.8	–	–
	KB1	492.0	162.0 ± 0.45	73.82	0.789	78.9
	KB2	480.0	71.10 ± 0.69	32.48	0.907	90.7

**Table 5:** Activation thermodynamic parameters for CS in the absence and presence of KB1 and KB2 at different temperatures.

Compounds	Arrhenius		Transition state			
	Slope	$\:{\text{E}}_{\text{a}}^{\text{*}},$ kJ mol^− 1^	Slope	Intercept	ΔH^*^, kJ mol^− 1^	− ΔS^*^, J mol^− 1^ K^− 1^
Blank	− 1237.3	23.69	− 1101.6	3.7695	21.09	125.39
KB1	− 1862.7	35.66	− 1727	4.9092	33.07	103.57
KB2	− 1271.5	24.35	− 1135.8	2.78441	21.75	144.26

### Adsorption process study

**Fig. 9:**
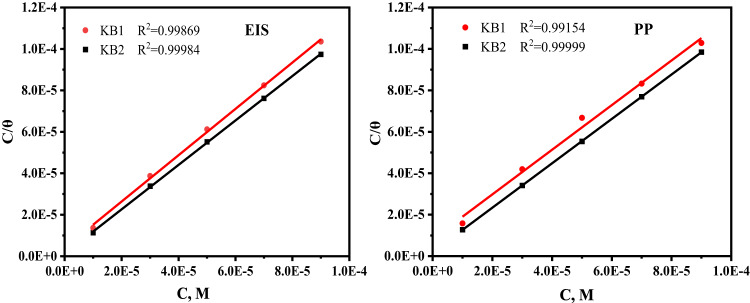
Langmuir isotherms for the synthesized KB1 and KB2 utilizing data obtained *via* EIS and PP methods at 298 K.

Studying adsorption isotherms is a potent tool for understanding the physicochemical interactions between corrosion inhibitors and metallic surfaces. Organic molecules inhibit corrosion by adsorbing onto the metal surface, displacing water molecules, and forming a protective barrier that blocks corrosive agents. In the H_2_SO_4_ solution, numerous models were studied to examine the connection regime at the inhibitors/CS interface and identify the best isotherm among the many adsorption isotherms^52,71^. The Langmuir model (Eq. (7)) demonstrated optimal fitting, as confirmed by linear C/θ versus C plots (Fig. 9) with slopes and regression coefficients (R²) approaching unity *via* EIS and PP approaches. This alignment validates the isotherm’s applicability for adsorption analysis^72^.

7 $$\:\frac{\text{C}}{{\uptheta\:}}=\frac{1}{{\text{K}}_{\text{a}\text{d}\text{s}}}+\text{C},$$.

where C represents the concentrations of KB1 and KB2, K_ads_ is the adsorptive equilibrium constant, and θ denotes the fractional surface covered by the employed inhibitors. K_ads_ can be computed from the intercept of Eq. (7). Equation (8) was utilized to obtain the standard free energy of adsorption ($\:{\triangle\text{G}}_{\text{a}\text{d}\text{s}}^{^\circ\:}$).

8 $$\:{\triangle\text{G}}_{\text{a}\text{d}\text{s}}^{^\circ\:}=-2.303\text{R}\text{T}\text{l}\text{o}\text{g}\left({\text{K}}_{\text{a}\text{d}\text{s}}\text{x}55.5\right),$$.

where 55.55 designates the concentration of water in solution (M). The values of Langmuir isotherm parameters are provided in Table 6. The tested inhibitor molecules have a substantial adsorption ability as implied by the high K_ads_ values, resulting in improved corrosion resistance^73^. According to Table 6’s data, $\:{\triangle\text{G}}_{\text{a}\text{d}\text{s}}^{^\circ\:}$ values are negative, demonstrating that the inhibitors under study are adsorbing spontaneously on the CS surface. Based on literature^74^, $\:{\triangle\text{G}}_{\text{a}\text{d}\text{s}}^{^\circ\:}$values below − 20 kJ mol^−1^ suggest physisorption, which happens when the inhibitor and the surface interact electrostatically. On the other hand, values close to or higher than − 40 kJ mol^−1^ indicate chemisorption, which denotes the creation of coordination bonds through electron sharing or transfer. The adsorption of KB1 and KB2 onto the CS surface primarily happens *via* the chemisorption mechanism, which is started by physical interactions, according to the computed $\:{\triangle\text{G}}_{\text{a}\text{d}\text{s}}^{^\circ\:}$values.

**Table 6:** Langmuir isotherm parameters for KB1 and KB2 at 298 K.

Compound	EIS				PP			
	Slope	*R* ^2^	K_ads_ x10^4^, M^− 1^	− ΔG^o^_ads_, kJ mol^− 1^	Slope	*R* ^2^	K_ads_x10^4^, M^− 1^	− ΔG^o^_ads_, kJ mol^− 1^
KB1	1.11795	0.99984	24.9	40.7	1.07697	0.99154	12.1	38.9
KB2	1.07232	0.99869	85.8	43.8	1.07113	0.99999	50.8	42.5

### Effect of immersion time

**Fig. 10:**
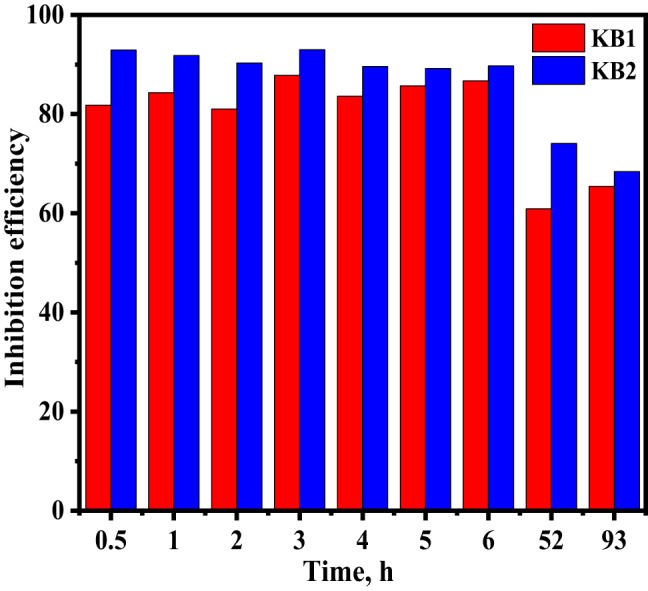
The impact of lengthy immersion periods on the effectiveness of KB1 and KB2 inhibition for CS dissolution in 0.5 M H_2_SO_4_ with 9 × 10^− 5^ M at 298 K.

EIS is a practical method for evaluating the inhibition process after extended immersion^75^. The CS surface was subjected to 0.5 M H_2_SO_4_ solution at different immersion times, without and with 9 × 10^− 5^ M of KB1 and KB2 at 298 K. The effects of KB1 and KB2 on CS corrosion in 0.5 M H_2_SO_4_ at various intervals are represented in Fig. 10. Table 7 provides the %IE values for different immersion times. The tested inhibitors demonstrated good stability and consistent inhibition performance, as indicated by the minimal change in %IE values over an extended immersion period of 6 h. All *R*_*ct*_ values were noticeably higher in the presence of KB1 and KB2 than in H_2_SO_4_ solution-free, suggesting that the adsorbed layer shields the CS from the harsh effects of the acidic medium^66,76^. The disintegration of the inhibitor film may have caused a decrease in inhibitory action after 6 h. This investigation further demonstrates the high stability and effectiveness of the tested inhibitors.

**Table 7:** EIS parameters for CS in 0.5 M H_2_SO_4_ without and with 9 × 10^− 5^ M of KB1 and KB2 at various immersion times.

Compound	Time, h	*R*_s_, Ω cm²	*R*_ct_, Ω cm²± SD	Goodness of fit(x10^− 3^)	θ	%IE
Blank	0.5	2.124	69.44 ± 1.69	5.528	–	–
	1	2.105	102.3 ± 1.44	5.424	–	–
	2	2.090	149.8 ± 2.08	5.673	–	–
	3	2.215	131.5 ± 2.16	4.560	–	–
	4	2.109	229.0 ± 1.76	5.487	–	–
	5	2.120	262.0 ± 1.69	5.381	–	–
	6	2.125	286.0 ± 1.80	5.183	–	–
	52	2.137	699.4 ± 1.31	4.691	–	–
	93	1.985	848.0 ± 1.67	6.993	–	–
KB1	0.5	2.750	382.3 ± 2.49	3.217	0.818	81.8
	1	2.741	652.7 ± 2.16	3.597	0.843	84.3
	2	2.717	787.8 ± 1.67	3.310	0.810	81.0
	3	2.684	1075.0 ± 1.59	3.634	0.878	87.8
	4	2.653	1393.0 ± 1.63	3.511	0.836	83.6
	5	2.630	1832.0 ± 2.74	3.813	0.857	85.7
	6	2.620	2150.0 ± 1.51	3.821	0.867	86.7
	52	2.641	1788.0 ± 1.68	3.043	0.609	60.9
	93	2.517	2453.0 ± 2.49	4.539	0.654	65.4
KB2	0.5	2.970	977.9 ± 1.59	3.997	0.929	92.9
	1	2.923	1245.0 ± 1.51	4.592	0.918	91.8
	2	2.897	1540.0 ± 2.94	5.014	0.903	90.3
	3	2.936	1890.0 ± 1.63	4.984	0.930	93.0
	4	2.936	2200.0 ± 1.45	5.152	0.896	89.6
	5	2.960	2420.0 ± 2.45	5.170	0.892	89.2
	6	2.945	2770.0 ± 2.49	5.269	0.897	89.7
	52	2.558	2700.0 ± 1.59	4.284	0.741	74.1
	93	2.568	2685.0 ± 1.40	4.463	0.684	68.4

### Zero charge potential

**Fig. 11:**
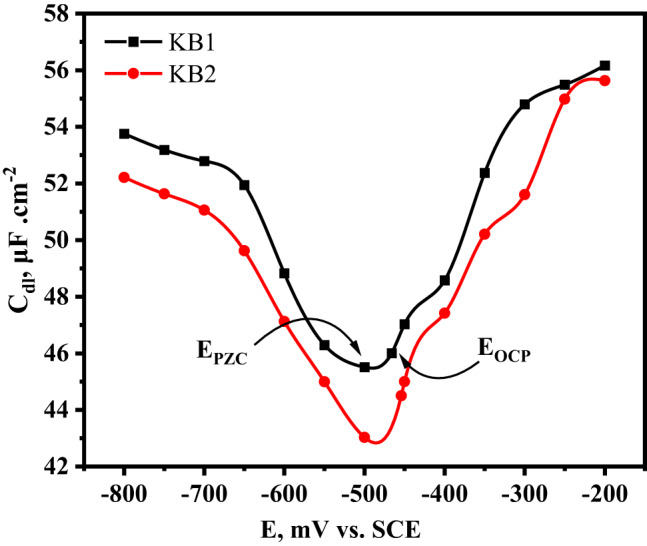
The plot of C_dl_ vs. E for CS in 0.5 M H_2_SO_4_ solution with 5 × 10^− 5^ M of KB1 and KB2 at 298 K.

The tested inhibitors are adsorbed from H_2_SO_4_ solution onto the CS surface *via* chemical and physical interactions. The adsorption of inhibitors begins with physical interactions. After protonated molecules adsorb, charge sharing or transfer takes place between the metal’s d orbitals and the molecules. Numerous factors, including the charge or dipole moment of the inhibitor molecules, the inhibitor’s chemical structure, and the charge of the CS surface, can potentially influence the adsorption process^77^. The PZC of the CS in the inhibited solution was defined *via* the EIS technique to obtain additional information regarding the surface charge of the CS. Figure 11 shows the dependence of double-layer capacitance (C_dl_) on the applied potential. The value of the CS’s E_PZC_ corresponds to the minimum value of C_dl_. The surface charge is determined by the position of E_ocp_ relative to the E_PZC_, and Eq. (9) was used to calculate the net surface charge of the CS^78^.

9 $$\:{\text{E}}_{\text{r}}={\text{E}}_{\text{o}\text{c}\text{p}}-{\text{E}}_{\text{p}\text{z}\text{c}}$$.

where E_r_ stands for Antropov’s ‘‘rational’’ corrosion potential. E_r_> 0 denotes a positive net charge of the CS surface at open circuit potential, while E_r_ <0 suggests a negative one. The CS surface was positively charged at the OCP when using KB1 and KB2, as implied by the positive E_r_ values for both inhibitors (Table 8). Due to electrostatic repulsion, protonated inhibitors find it challenging to approach the positively charged CS surface. On the other hand, adsorption of the SO_4_^2−^ anion in aqueous H_2_SO_4_ solution is preferable since it generates an excess of negative charges. Nevertheless, because the SO_4_^2−^ anion is more hydrated than Cl^−^ anion, it adsorbs poorly on the positively charged CS surface, leaving more active sites available for the inhibitors to adsorb directly onto the charged metal surface. Also, it is crucial to remember that protonated species of KB1 and KB2 may be discharged on the CS surface, so neutral molecules that result may be able to adsorb on the surface of CS following chemisorption mechanisms *via* the unshared pair of electrons on the heteroatoms (N and S) to reduce metal disintegration by providing a protective chemisorbed layer^52^.

**Table 8:** Values of E_ocp_, E_PZC,_ and E_r_ recorded for CS in 0.5 M H_2_SO_4_ solution with 5 × 10^− 5^ M of KB1 and KB2 at 298 K.

Inhibitors	E_ocp_ (mV vs. SCE)	E_PZC_ (mV vs. SCE)	E_*r*_ (mV vs. SCE)
KB1	− 466	− 500	34
KB2	− 454	− 500	46

### Surface characterization

#### AFM analysis

**Fig. 12:**
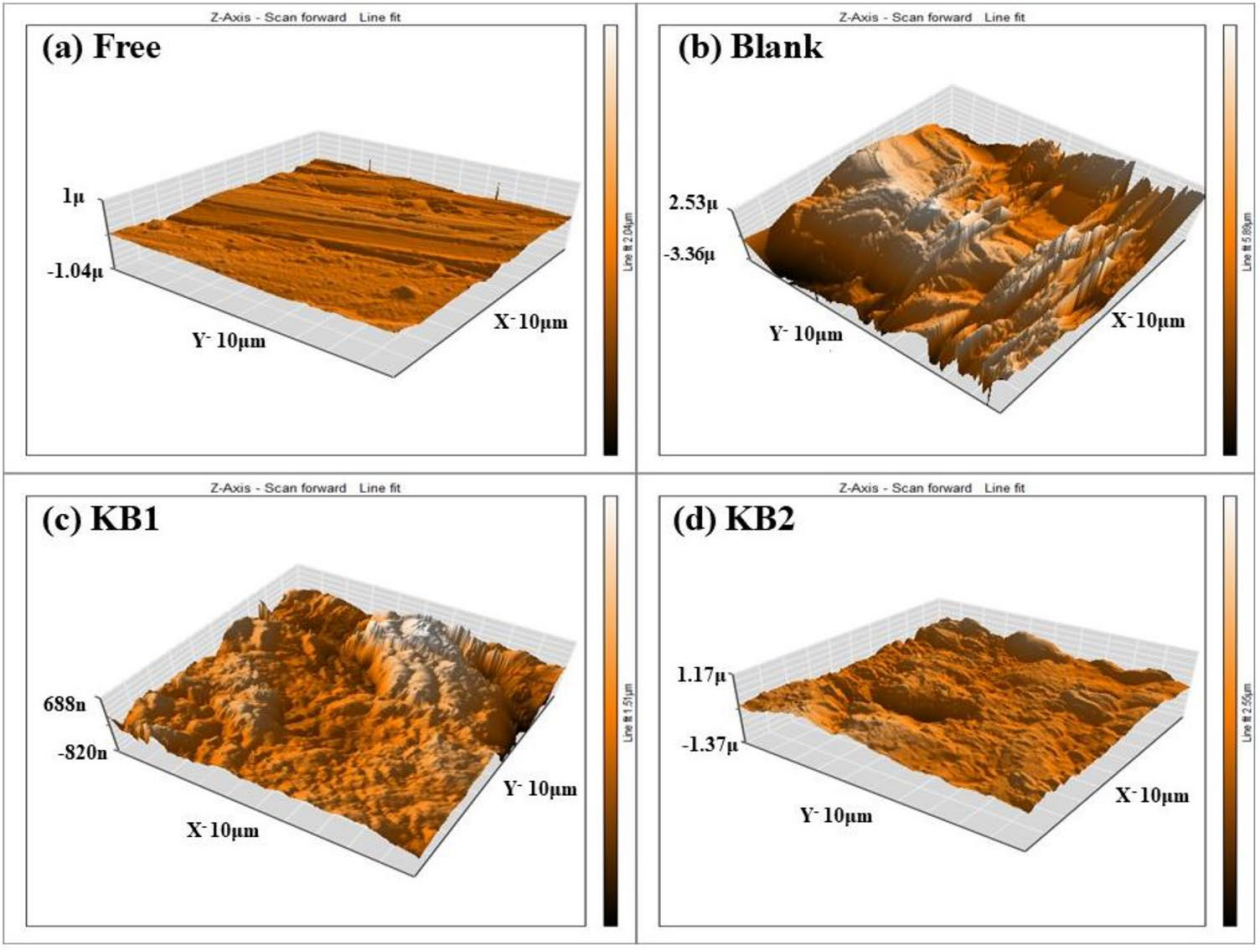
3D AFM images of the surface of polished CS (**a**), following immersion in 0.5 M H_2_SO_4_ solution before (**b**) and after the addition of KB1(**c**), and KB2 (**d**) for one day at 298 K.

Metal surface roughness can be evaluated using AFM analysis^79^. To test the compounds’ ability to impede corrosion, the CS coupons were submerged in the blank solution for 24 h at 298 K without and with 9 × 10^− 5^ M of KB1 and KB2. Figure 12a–d depicts three-dimensional AFM images of the CS surfaces. Before the CS immersion in 0.5 M H_2_SO_4_, the CS’s surface appeared smooth with a roughness value of 34.16 nm (Fig. 12a), following immersion in 0.5 M H_2_SO_4_ (Fig. 12b), the surface was damaged and became rougher and the roughness value increased to 853.81 nm, adding KB1 and KB2 made the surface smoother and the roughness values diminished to 153.88 nm and 114.65 nm, respectively when related to the blank sample (Fig. 12c and d). The lower roughness value for KB2 indicates that it protects the CS more effectively than KB1 in 0.5 M H_2_SO_4_ by adhering to the CS surface and forming a protective film^80,81^.

#### XPS analysis

**Fig. 13:**
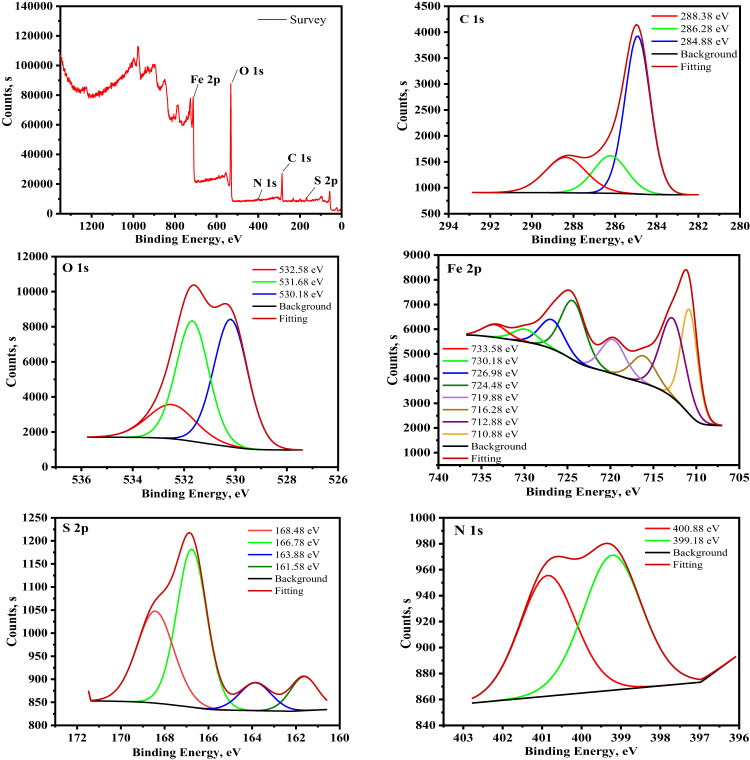
XPS spectra of the adsorbed layer of KB1 developed on the CS surface following immersion in 0.5 M H_2_SO_4_.

**Fig. 14:**
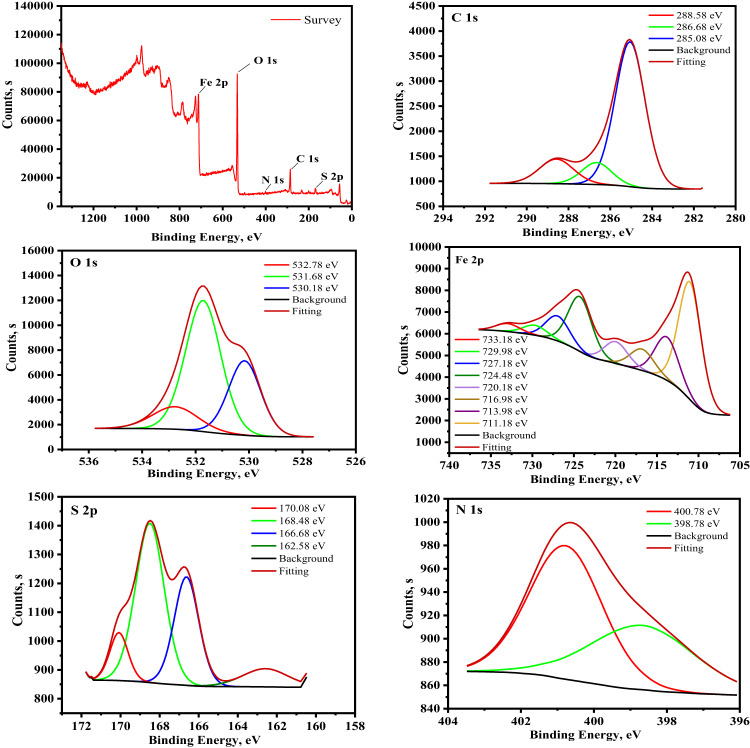
XPS spectra of the adsorbed layer of KB2 developed on the CS surface after immersion in 0.5 M H_2_SO_4_.

The surface phenomena were examined using XPS to fully comprehend the elements of the adsorbed layer that developed on the exposed portion of CS submerged in 0.5 M H_2_SO_4_ solution with 9 × 10^− 5^ M of KB1 and KB2 after immersion for 24 h, as indicated in the acquired XPS spectra in Figs. 13 and 14^82^. The binding energies (BE, eV) with the assignment for each peak component are indicated in Table 9. As depicted in Figs. 13 and 14, the spectra comprise C 1s, N 1s, O 1s, Fe 2p, and S 2p, supporting the adsorption of KB1 and KB2 on the CS surface. Three peaks were visible in the C1s XPS spectra for KB1 and KB2. The C–H, C–C, and C = C bonds may cause the first peak, which is located at 284.88 and 285.08 eV^83^. The C–N bonds may be connected to the second peak, which is located at 286.28 and 286.68 eV, while the last peaks, at 288.38 and 288.58 eV, may be associated with C = N bonds, which are included in the structures of KB1 and KB2 and confirm their adsorption^84^. The XPS spectra of O 1s exhibited three peaks (Figs. 13 and 14). The first and second peaks, observed at 530.18 and 531.68 eV respectively, correspond to Fe_2_O_3_ / Fe_3_O_4_ and FeOOH. The third peak, at 532.58 and 532.78 eV, is associated with adsorbed water molecules^85^. XPS spectra of Fe 2p exhibit seven distinct peaks (Figs. 13 and 14). Metallic iron is characterized by peaks at 707.29 eV and 711.24 eV. The Fe^3+^ state is identified at 710.88 and 711.18 eV, while Fe^3+^ is at 712.88 and 713.98 eV. The 716.28 and 716.98 eV peaks may be related to Fe 2p_3/2_ of Fe^2+^. Additionally, satellite peaks of Fe³^+^ are observed at 719.88 and 720.18 eV. The Fe 2p_1/2_ component of Fe^2+^ appears at 724.48 eV, whereas Fe 2p_1/2_ of Fe^3+^ exhibits peaks at 726.98 and 727.18 eV. Finally, the Fe 2p_1/2_ component of Fe^2+^ is detected at 730.18, 729.98, 733.58, and 733.18 eV^58^. Nitrogen’s XPS spectra (Figs. 13 and 14) showed two peaks, the first at 399.18, 398.78 eV, and the second at 400.88, 400.78 eV, which stand for N-H/N-H_2_, N-Fe, N–N^86^and C = N–N bonds^87^respectively. Four peaks for S2p appeared in the XPS spectra (Figs. 13 and 14). The first at 161.58, 162.58, and 163.88 eV stand for sulphide and S-C bonds, while the second at 166.78, 166.68, 168.48, and 170.08 eV ascribed to S-Fe bonds^87^. Based on the XPS data, the adsorption of KB1 and KB2 on CS was proved.

**Table 9:** The binding energies (eV) with their assignments for the CS after being immersed in 0.5 M H_2_SO_4_ containing 9 × 10^− 5^ M of KB1 and KB2.

Core element	0.5 M H_2_SO_4_ + KB1		0.5 M H_2_SO_4_ + KB2	
	BE, eV	Assignments	BE, eV	Assignments
C 1s	284.88	C–H, C–C, and C = C	285.08	C–H, C–C, and C = C
	286.28	C–N	286.68	C–N
	288.38	C = N	288.58	C = N
O 1s	530.18	Fe_2_O_3_ / Fe_3_O_4_ oxide	530.18	Fe_2_O_3_ and/or Fe_3_O_4_ oxide
	531.68	FeOOH	531.68	FeOOH
	532.58	Adsorbed water molecules	532.78	Adsorbed water molecules
Fe 2p	710.88	Metallic iron	711.18	Metallic iron
	712.88	Fe^3+^	713.98	Fe^3+^
	716.28	Fe 2p_3/2_ of Fe^2+^	716.98	Fe 2p_3/2_ of Fe^2+^
	719.88	Satellite of Fe^3+^	720.18	Satellite of Fe^3+^
	724.48	Fe 2p_1/2_ of Fe^2+^	724.48	Fe 2p_1/2_ of Fe^2+^
	726.98	Fe 2p_1/2_ of Fe^3+^	727.18	Fe 2p_1/2_ of Fe^3+^
	730.18, 733.58	Fe 2p_1/2_ of Fe^2+^	729.98, 733.18	Fe 2p_1/2_ of Fe^2+^
N 1s	399.18	N–H/N-H_2_, N–Fe, N–N	398.78	N–H/N-H_2_, N–Fe, N–N
	400.88	C = N–N	400.78	C = N–N bond
S 2p	161.58,163.88	Sulphide, S–C	162.58	Sulphide, S–C
	166.78,168.48	S–Fe	166.68,168.48,170.08	S–Fe

#### FT-IR analysis

**Fig. 15:**
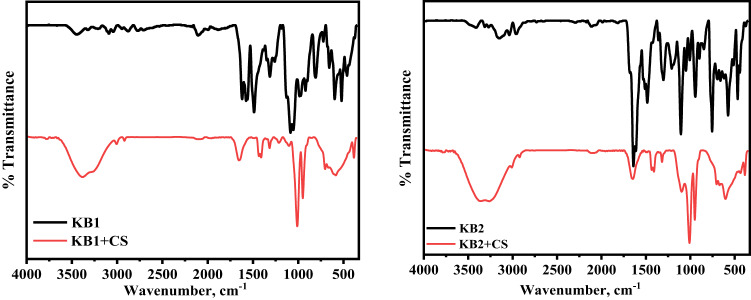
FT-IR spectra of the pure inhibitor and the layers that developed on CS samples after one day of immersion in 0.5 M H_2_SO_4_ with 9 × 10^− 5^ M of inhibitors at 298 K.

Figure 15 presents the utilization of infrared spectroscopy to confirm the interaction among the inhibitors and the CS surface in 0.5 M H_2_SO_4_. The comparison of spectra for pure inhibitors and the layers that developed on CS samples show that the inhibited CS coupons resemble that of the free inhibitors, with shifts observed in the spectra, likely due to the interaction among inhibitor molecules and the CS surface, offering definitive evidence of inhibitor adsorption on the metal surface, thereby facilitating corrosion protection^88^. The shifting and removal of some peaks in the spectra after immersion indicated that the inhibitors effectively adsorbed onto the surface of CS *via* functional groups, resulting in inhibition^89,90^.

### UV–Visible spectra

**Fig. 16:**
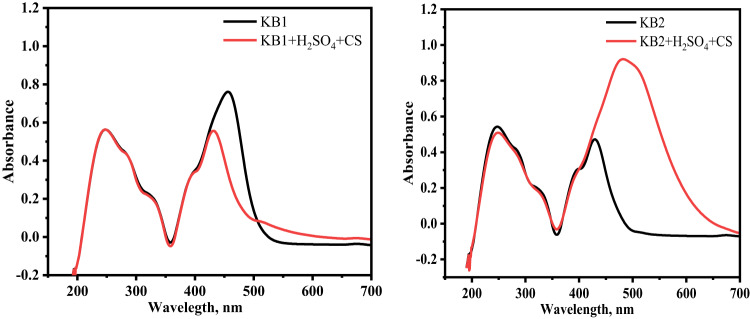
UV–visible spectra for test solutions of KB1 and KB2 before (black color) and after (red color) dipping CS for 48 h at 298 K.

To verify the potential for developing the inhibitor-Fe complex, Fig. 16 displays the UV-Visible absorption spectra recorded for the test solutions of 9 × 10^− 5^ M of KB1 and KB2 before and after 48 h of CS immersion at 298 K. Before CS immersion, absorption bands for KB1 and KB2 are visible in the spectra at (248 nm, 456 nm) and (247 nm, 429 nm), respectively, which are ascribed to π‒π*and n‒π* possessing a significant charge transfer characteristic^91,92^. However, after CS immersion, the spectra exhibited changes in the location of absorption bands. The KB1 spectrum displayed two distinct bands at 247 nm and 431 nm, while the observed bands for KB2 appeared at 249 nm and 482 nm. The alteration in the absorption maximum (λ_max_) and/or the shift in absorbance values indicate the development of a complex between the tested inhibitor molecules and the Fe^2+^ ions in solution, as reported in literature^93^. These experimental results provide strong evidence for the formation of a protective metal/inhibitor complex film on the CS, confirming the complexation between Fe^2+^ and KB1/KB2 in H_2_SO_4_ solution^94^.

### Theoretical calculations

#### DFT

**Fig. 17:**
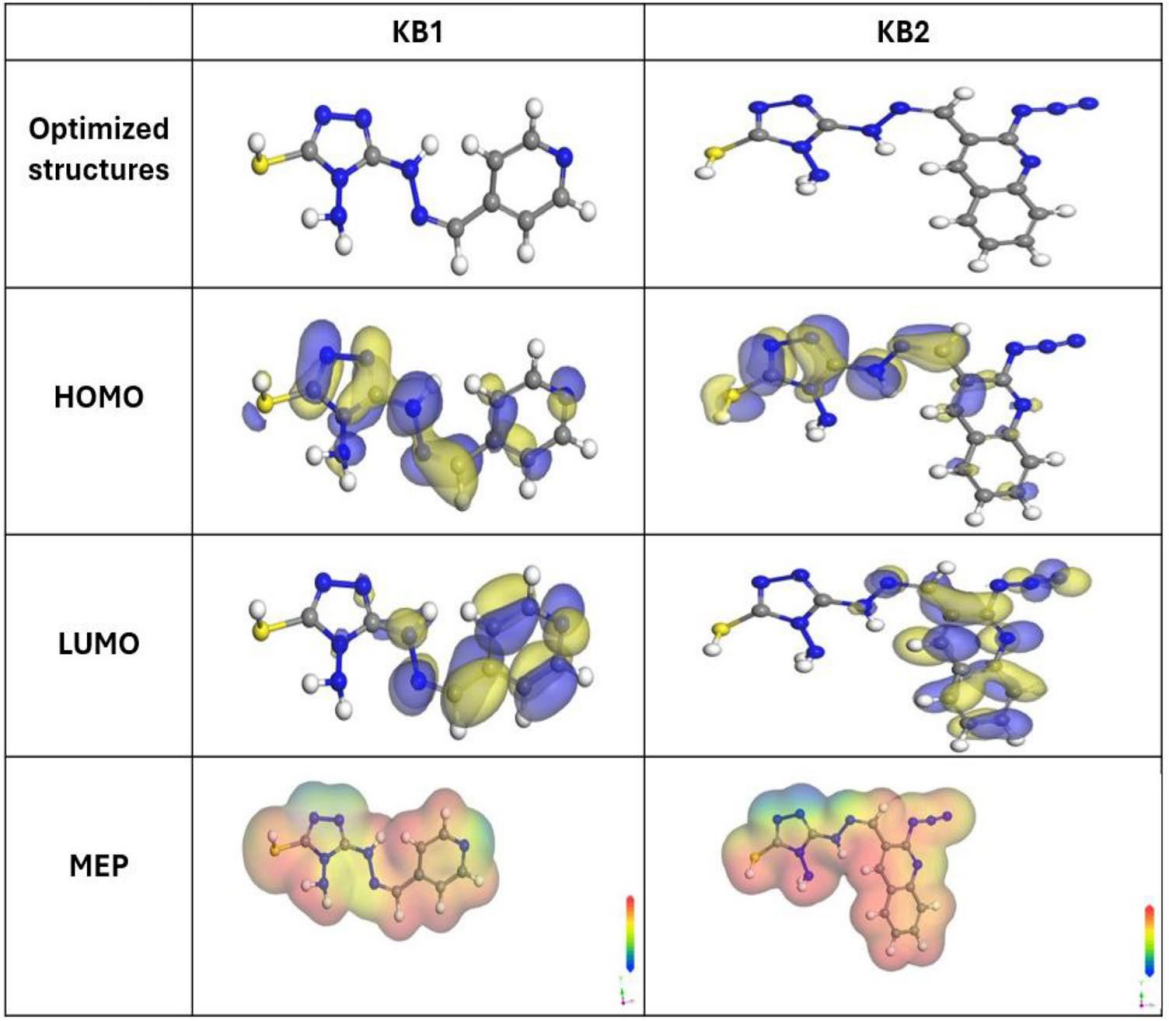
The optimized structures, HOMO, LUMO, and MEP of the KB1 and KB2 using DMol^3^ module.

Figure 17 presents the optimized structures alongside the spatial distributions of the highest occupied molecular orbital (HOMO), the lowest unoccupied molecular orbital (LUMO), and molecular electrostatic potential (MEP) mapping for the KB1 and KB2 molecules in an aqueous environment. The corresponding theoretical parameters are provided in Table 10. Based on Frontier Molecular Orbital (FMO) theory, the interaction between a molecule and a metallic surface can be elucidated through the analysis of HOMO and LUMO energy levels, which offer valuable insight into the molecule’s electron-donating and -accepting capabilities. These energy levels determine whether a molecule exhibits anticorrosive properties^95^. Higher HOMO energy and lower LUMO energy generally correlate with enhanced anticorrosive performance. KB2 molecules exhibit a higher E_HOMO_ (− 4.98 eV) than KB1 molecules (− 5.11 eV), according to the findings in Table 10, indicating that KB2 possesses superior corrosion inhibition capabilities. It is evident from Fig. 17 that the HOMO orbital for KB1 and KB2 concentrated on the triazole, hydrazone, pyridine, and quinoline moieties, rendering these areas more vulnerable to electrophilic assaults on the CS. The findings of the experiments support the idea that the tested inhibitor molecules can efficiently adhere to the CS, strengthening their protective impact against corrosion. Compared to the KB1 molecules, which have an E_LUMO_ value of − 2.26 eV, the KB2 molecules exhibit a lower E_LUMO_ (− 2.48 eV), suggesting that KB2 has a greater propensity for adsorption than KB1. Because it is desirable for enabling the transport of an electron from the inhibitor to the metallic surface, reducing the energy gap (ΔE) is essential for increasing the effectiveness of corrosion inhibitors^96^. In Table 10, KB2 has ΔE of 2.50 eV, significantly lower than the ΔE of KB1 (2.85 eV), demonstrating the increased susceptibility for the adsorption of KB2 onto CS. Corrosion inhibitors often possess low electronegativity (χ) values, reflecting their ability to donate electrons to the metal, thereby enhancing their protective performance^97^. However, the inhibitor molecule can receive the electron from the metal surface if its χ value is high. It occurs through back-donation, which enhances the bond at the steel interface^98^. The molecules of KB1 and KB2 have relatively high χ values, which facilitate the back-donation of electrons and promote the formation of more stable interactions with the CS, as detailed in Table 10. Furthermore, the reactivity and stability of inhibitor molecules can be evaluated through their global softness (σ) and hardness (η) parameters. The smooth passage of electrons to the CS *via* adsorption gives the soft molecules greater protective capacity than the hard molecules, rendering them effective corrosion inhibitors^99^. Table 10 illustrates that the KB2 molecule has better inhibitory properties than the KB1 molecule because of its greater σ and lower η values. Additionally, the number of electrons transferred (∆N) and ∆E_back−donation_ are pivotal factors in assessing the inhibitor’s capacity to give or accept electrons. It is suggested that electron transfer from the inhibitor to the metal occurs if ∆N values are higher than 0. Conversely, electron transfer from the metal to the inhibitor molecule is feasible if ∆N values (< 0)^100^. The molecules of KB1 and KB2 have positive ∆N values, which indicate their ability to supply electrons to the CS, as recorded in Table 10. Moreover, the ∆E_back−donation_ becomes less than zero when η > 0, indicating that electrons are moved from the metal to the inhibitor and then donated back to the metal. This dynamic process is desirable^101^. Table 10 presents negative ∆E_back−donation_ values for KB1 and KB2, suggesting their strong tendency for back-donation and the development of robust bonds^47^. The dipole moment (µ) indicates how polarizable the tested inhibitors are. A high value of µ suggests strong polarizability, which in turn shows high reactivity, prompting inhibitors to adhere to the surface firmly^102^. As mentioned in Table 10, KB2 has a higher dipole moment value (13.29 Debye) than KB1 (6.93 Debye), emphasizing its higher tendency to be adsorbed onto the CS, as well as strengthening its inhibitory impact. A noticeable correlation was observed among the molecular surface area of the tested inhibitors and their ability to safeguard the CS from dissolution. A larger molecular surface area enhances the inhibition efficiency and increases the contact area on the CS^103^. As presented in Table 10, KB2 exhibited a larger molecular surface area (329.54 Å²), resulting in greater inhibition efficiency when compared to KB1(252.02 Å²). The inhibitors’ active sites may be investigated *via* MEP mapping, a three-dimensional visual depiction that uses a molecule’s general charge distribution to determine its net electrostatic effect^104^. The red areas in Fig. 17 indicate the highest electron density, where the MEP is highly negative (nucleophilic reaction). In the meantime, the blue areas indicate the highly positive areas (electrophilic reaction)^103^. The highly negative regions in Fig. 17 are centered over free nitrogen, sulphur, pyridine, and quinoline moieties. In contrast, the highly positive areas over the triazole ring’s allocated nitrogenous atoms are due to the hybridization of lone pairs allocated on the nitrogenous atoms at positions (1,2) is the sp^2^ orbital, which is allocated in the same plane of the ring and does not overlap with other p orbitals of the ring. The areas with higher electron densities (shown by the red area) for KB1 and KB2 molecules are probably the most desirable for interactions with the CS, resulting in the configuration of sustainable protective layers.

**Table 10:** Quantum chemical parameters for KB1 and KB2.

Quantum parameters	KB1	KB2
E _HOMO_, eV	− 5.11	− 4.98
E _LUMO_, eV	− 2.26	− 2.48
∆E = E _LUMO_− E _HOMO_, eV	2.85	2.50
I, eV	5.11	4.98
A, eV	2.26	2.48
χ, eV	3.68	3.73
η, eV	1.42	1.25
σ, eV	0.70	0.80
∆N, eV	1.16	1.31
∆E_back−donation,_ eV	− 0.36	− 0.31
Dipole moment value, debye	6.93	13.29
Molecular surface area, A^o2^	252.02	329.54

#### MC simulation

**Fig. 18:**
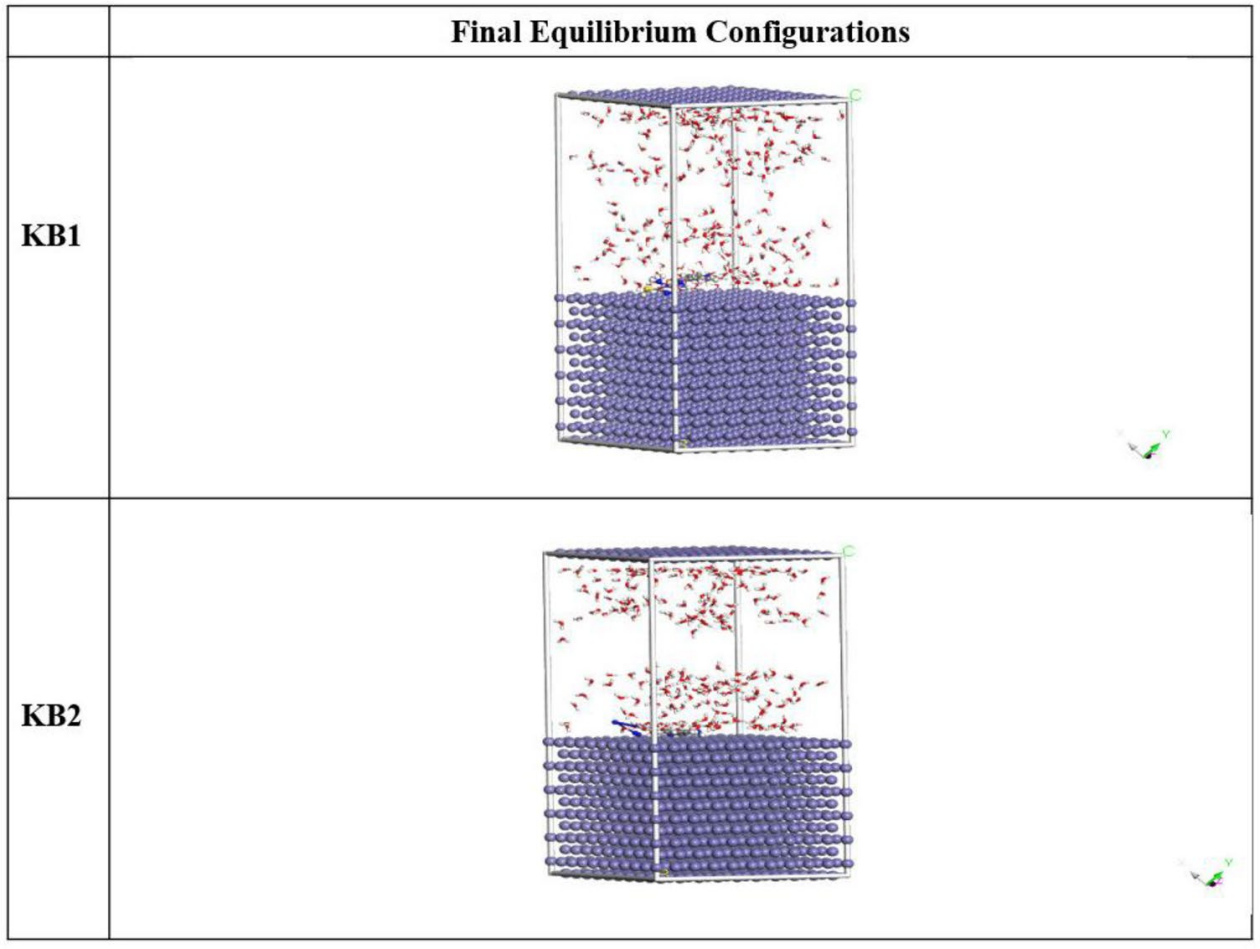
The most suitable configuration for the adsorption of KB1 and KB2 on Fe (11 0).

The best adsorption configurations and interactions between the tested inhibitors and the CS surface were investigated *via* MC simulations^105^. The most desirable adsorption configurations of KB1/KB2 are displayed in Fig. 18. KB1 and KB2 molecules are nearly parallel to the Fe (110) surface as a result, CS in the corrosive solution can be kept from corroding as much as possible *via* parallel adsorption which optimizes the surface-coverage characteristics of the inhibitors^106^. The adsorption energies computed *via* MC in Table 11 demonstrate significantly stronger adsorption for KB2, evidenced by its more negative adsorption energy (− 3083.62 kcal/mol) compared to KB1 (− 3030.47 kcal/mol). These results support the idea that KB2 could form a firmer adsorptive layer on the CS, enhancing corrosion resistance^107^. Furthermore, Table 11 asserts a higher inhibition proficiency for KB2 than KB1 by demonstrating that the adsorption energies values of KB2 for the pre-geometry optimization step, or unrelaxed (− 3240.13 kcal mol^− 1^), are more negative than KB1 (− 3181.06 kcal mol^− 1^) and for the post-geometry optimization step, or relaxed (156.51 kcal mol^− 1^, respectively), are higher than KB1 (150.59 kcal mol^− 1^). If water molecules or adsorbed inhibitors have been removed, the dE_ads_/dN_i_ values provide insight into the metal-adsorbates configuration energy^108^. In Table 11, the dE_ads_/dN_i_ value for KB2 is much higher (− 172.48 kcal mol^− 1^) than the KB1 molecule (− 142.41 kcal mol^− 1^). Accordingly, the KB2 molecule has a higher propensity for adsorption than the KB1 molecule. Furthermore, the dE_ads_/dN_i_ values for water are low (about − 7.65 kcal mol^− 1^) when compared to KB1 and KB2 values, indicating that inhibitor molecules adsorbed more robustly than water molecules, reinforcing the replacement of water molecules *via* tested inhibitor molecules^109^. Due to this replacement, a coherent protective layer developed onto the CS in the corroding medium. These computational findings are consistent with the observed experimental data on corrosion resistance.

**Table 11:** The MC simulation data for the adsorption of the KB1 and KB2 on Fe (110).

Structures	Adsorption energy/kcal mol^− 1^	Rigid adsorption energy/kcal mol^− 1^	Deformation energy/kcal mol^− 1^	dE_ads_/dN_i_: inhibitor, kcal mol^− 1^	dE_ads_/dN_i_: water, kcal mol^− 1^
Fe (1 1 0) KB1 Water	− 3030.47	− 3181.06	150.59	− 142.41	− 7.75
Fe (1 1 0) KB2 Water	− 3083.62	− 3240.13	156.51	− 172.48	− 7.54

### Corrosion Inhibition mechanism

**Fig. 19:**
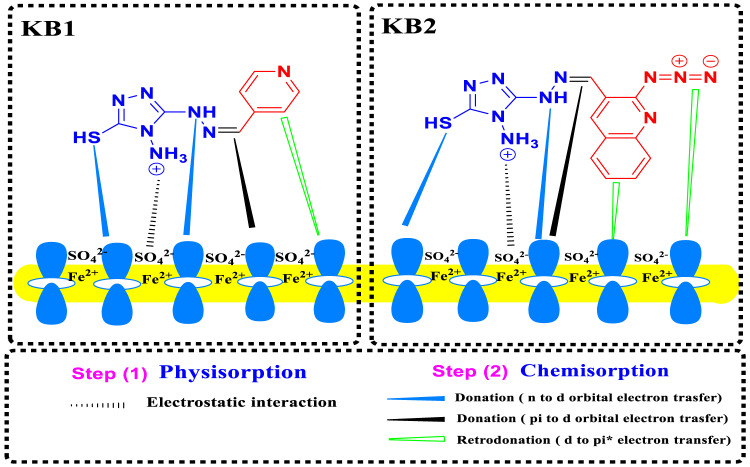
A schematic representation of the corrosion inhibition mechanism for CS in 0.5 M H_2_SO_4_.

Considering the theoretical and experimental results, Fig. 19 depicts the possible mechanism of corrosion inhibition for CS in 0.5 M H_2_SO_4_. According to the literature^110^, the adsorption between the inhibitor and the CS surface can occur physically, chemically, or both. Physical, which results from electrostatic interactions between the charged CS surface and the protonated groups of organic molecules^111^. In this context, the protonated inhibitors in H_2_SO_4_ can be adsorbed on CS surfaces through interaction with the negatively charged surface (caused by the adsorbed SO_4_^2−^ ions on it), resulting in physical adsorption. On the other hand, there are two strategies to achieve the chemical adsorption of the tested inhibitors on the CS surface through donor-acceptor interaction^90^. The first involves the interaction of π-electrons on the nitrogen atoms and π-electrons of the aromatic moieties and the vacant d-orbital of the CS, inhibiting it from dissolving in the corrosive medium. The second is accomplished by the formation of the Fe-inhibitor complex, which results from the chemical coordination of iron atoms as electron acceptors and the inhibitor’s heteroatoms as electron donors. Retro-donation, in which orbital electrons are transferred from the metal atoms to the inhibitor molecules’ antibonding molecular orbitals, is another way that inhibitor molecules might attach to metal surfaces^112^. The %IE of these derivatives is in the order KB2 > KB1, which is explained by the presence of quinoline moiety and azido group in the structure of KB2. Also, it has a lower energy gap and a higher softness value, which increases the availability of donation and retro-donation, thus improving and strengthening its adsorption on the CS surface.

### Comparative studies with previous reports

Notably, our compounds exhibited high inhibition efficiencies even at relatively low concentrations, whereas many literature-reported inhibitors required significantly higher concentrations to achieve comparable efficiency values, as indicated in Table 12. This demonstrates both the cost-effectiveness of KB1 and KB2 as well as their improved corrosion inhibition capabilities, as lower amounts are needed to protect the surface of CS. We think that this comparison gives an improved overview of the economic and practical benefits of our eco-friendly inhibitors.

**Table 12:** Comparison of studied inhibitors with some reported Triazole derivatives.

Inhibitor	Corrosive medium	Conc. of inhibitor (mol/L)	% IE		Sample	Reference
			PP	EIS		
4-Amino-1-((8-hydroxyquinolin-5-yl)methyl)-1,2,3-triazole-5-carboxylate (MHTC)	1.0 M HCl	10 ^− 3^	91	87	carbon steel	^113^
5-((Furan-2-yl)methyleneamino)-2*H*-1,2,4-triazole-3-thiol (FMT)	1.0 M HCl	5.0 × 10^− 3^	86	90	Mild steel	^114^
5-((Thiophen-2-yl)methyleneamino)-2*H*-1,2,4-triazole-3-thiol (TMT)			90	93		
4-Amino-1,2,4- triazole-3-thiol (ATT)	1.0 M H_2_SO_4_	1.0 × 10^− 3^	98.56	94.82	Mild Steel	^115^
4-Amino-5-methyl-1,2,4-triazole-3-thiol (AMTT)			99.39	95.72		
4-Amino-5-ethyl-1,2,4- triazole-3-thiol (AETT)			99.55	95.89		
Ethyl4-amino-1-((8-hydroxyquinolin-5-yl)methyl)-1*H*-1,2,3-triazole-5-carboxylate (EHTC)	0.5 M H_2_SO_4_	10^− 3^	95.5	94.1	carbon steel	^116^
4-Amino-1-((8-hydroxyquinolin-5-yl)methyl)- 1*H*-1,2,3-triazole-5-carbonitrile (AHTC)			95.1	93.6		
Methyl 4-amino-1-((8-hydroxyquinolin-5-yl)methyl)-1*H*-1,2,3-triazole-5- carboxylate (MHTC)			94.1	93.9		
5-Octylsulfanyl-1,2,4-triazole (TR8)	1.0 M HCl	10^− 3^	84.41	88.02	Mild Steel	^117^
5-Decylsulfanyl-1,2,4-triazole (TR10)			85.60	92.00		
4-Amino-3-(N-phthallimidomethyl)-1,2,4-triazole-5- thione (Tz-1)	1.0 M HCl	2.5 × 10^− 4^	95.36	95.16	Mild steel	^118^
3-(N-Phthalimidomethyl)-4-(4-pyridine)amino-1,2,4-triazole-5-thione (Tz-2)			92.45	93.82		
1-((1*H*-1,2,4-triazol-3-ylimino)methyl) naphthalen-2-ol (TMN)	1.0 M HCl	10 × 10^− 4^	88.87	91.12	carbon steel	^119^
N-(Furan-2-ylmethylene)-1*H*-1,2,4-triazol-3-amine (FTA)			80.46	84.65		
N-(Thio phen-2-ylmethylene)-1*H*-1,2,4-triazol-3-amine (TTA)			80.51	86.19		
4-Amino-5-phenyl-4*H*-1, 2, 4,- triazole-3-thiol (APTT)	1.0 M HCl	5.72 × 10^− 4^	94.32	96.04	Mild Steel	^120^
4-Amino-5-(2-hydroxy) phenyl-4*H*-1, 2, 4,-triazole-3-thiol(AHPTT)			93.05	95.40		
4-Amino-5-styryl-4*H*-1, 2, 4,-triazole-3-thiol, (ASTT)			96.45	97.03		
(*Z*)-4-Amino-5-(2-(pyridin-4-ylmethylene)hydrazinyl)-4*H*-1,2,4-triazole-3-thiol (KB1)	0.5 M H_2_SO_4_	9 × 10^− 5^	87.50	86.90	CS	Our work
(*Z*)-4-Amino-5-(2-((2-azidoquinolin-3-yl)methylene)hydrazinyl)-4*H*-1,2,4-triazole-3-thiol (KB2)			91.40	92.40		

## Conclusion

1. In this study, we synthesized and characterized two triazole compounds, KB1 and KB2. These compounds were then tested for their ability to impede dissolution in 0.5 M H_2_SO_4_ *via* different electrochemical techniques (OCP, PP, and EIS), surface characterizations, UV-Visible spectroscopy, and theoretical investigations.
2. The results showed that both KB1 and KB2 inhibitors effectively impeded the corrosion of CS in the acidic medium, with KB2 exhibiting the highest protection efficiency of 92.4% at a dose of 9 × 10^-5^ M.
3. The tested compounds were identified as mixed-type inhibitors *via* the PP measurement.
4. The addition of KB1 and KB2 results in a decrease in double-layer capacitances relative to the blank solution. This validates their adsorption on the CS.
5. Analysis of the inhibitors’ adsorption behavior revealed that they aligned with the Langmuir adsorption isotherm. According to thermal studies (298–328 K), strong adsorptive interactions with the CS are indicated by the inhibitors’ improved inhibition at 318 K (88.9% for KB1 and 93.6% for KB2), then decreased at 328 K, which implies that higher temperatures (328 K) may cause the desorption of KB1 and KB2 from the CS.
6. The slight change in %IE value during a long immersion period extended to 6 h implies the stability of the protective film developed by KB1 and KB2 onto the CS. To investigate the inhibitory mechanism, ZCP measurements were employed, which demonstrate that the inhibitors first engaged in electrostatic interaction with the positively charged CS surface. After this, robust chemical bonds are formed.
7. Surface characterization techniques (AFM, XPS, and FT-IR) verified that the CS surface had a protective film that successfully protected it from the H_2_SO_4_ environment. The potential for complex establishment between ferrous ions and the tested inhibitors was validated *via* UV-visible spectroscopy.
8. DFT and MC simulations have explored the molecular structure, reactivity, and adsorption configuration of KB1 and KB2. They validated the placement of their multi-active sites and supported the experimental findings. The data acquired in this report highlights the potential application of synthesized triazoles as potent inhibitors against dissolution in metal treatment processes.

## Electronic supplementary material

Below is the link to the electronic supplementary material.

Supplementary Material 1

## Data Availability

All data generated or analysed during this study are included in this published article (and its Supplementary Information files).
