# Supplementary material for: A comprehensive experimental and theoretical perspective of novel triazole-based pyridine and quinoline derivatives for corrosion protection of carbon steel in sulfuric acid solution
Source: Sci Rep. 2025 Jul 24;15:26938. doi: 10.1038/s41598-025-10619-5 (PMC12289913; doi:10.1038/s41598-025-10619-5)
Supplement: Supplementary file 1 — Supplementary Material 1 [file 41598_2025_10619_MOESM1_ESM.docx]

**A comprehensive experimental and theoretical perspective of novel triazole-based pyridine and quinoline derivatives for corrosion protection of carbon steel in sulfuric acid solution**

Kamelia Belal^1,2^, A.H. El-Askalany^1^, Eslam A Ghaith^1,3^, Ahmed Fathi Salem Molouk^*1,2,3^

^1^Department of Chemistry, Faculty of Science, Mansoura University, Mansoura 35516, Egypt

^2^ Mansoura University Sustainable Energy Research Lab (MSER), Faculty of Science, Mansoura University, Mansoura 35516, Egypt

^3^Faculty of Science, New Mansoura University, New Mansoura City, Egypt

^*^ Correspondence author: Ahmed Fathi Salem Molouk

E-mail address: [molouk82@mans.edu.eg](mailto:molouk82@mans.edu.eg)

Supplementary Material

**General experimental:**

Melting points (uncorrected) were measured using a Gallenkamp melting-point apparatus and were uncorrected. The reaction mixture was monitored using thin-layer chromatography (TLC), which was performed on silica gel 60 F_254_ precoated aluminum sheets and visualized under ultraviolet (UV) light. Also, Infrared (IR) spectra were recorded on a JEOL FT-IR Spectrometer (Reflectance). A Bruker 400 M*Hz* or JEOL 500 M*Hz* spectrometers were used to record ^1^H-NMR and ^13^C-NMR spectra in the presence of DMSO-*d_6_* as solvent. Electron impact mass spectra were determined at 70 eV on a Varian MAT 3311 Kratos instrument (Micro-analytical center, Faculty of Science, Cairo University. Sonication was performed in a “Spectra lab model UCB 40D Ultrasonic cleaning bath” with a frequency of 40 kHz and power of 250 W. All chemicals and solvents were used as received from Sigma Aldrich and Fisher Scientific companies

Figure caption

**S1**. ^1^H-NMR spectrum of compound KB1.

**S2**. ^13^C-NMR spectrum of compound KB1.

**S3**. Mass spectrum of Compound KB1.

**S4**.^1^H-NMR spectrum of compound KB2.

**S5**. ^13^C-NMR spectrum of compound KB2.

**S6**. Mass spectrum of Compound KB2.


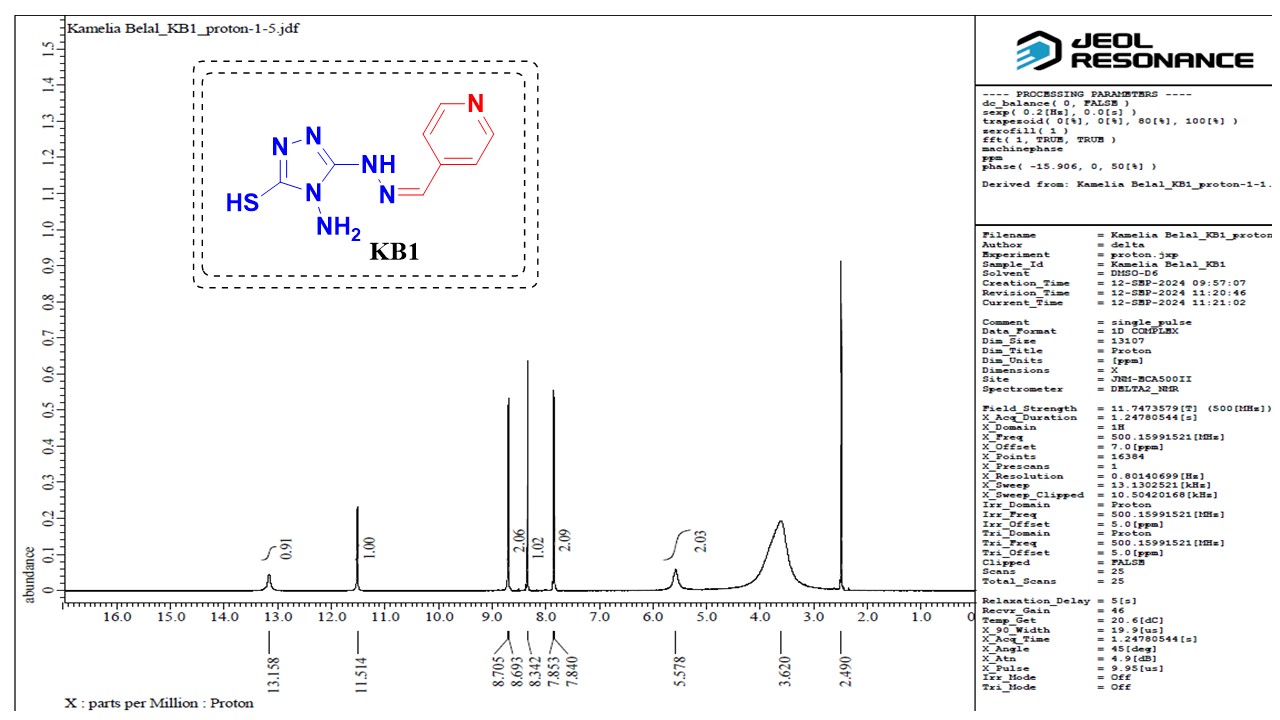


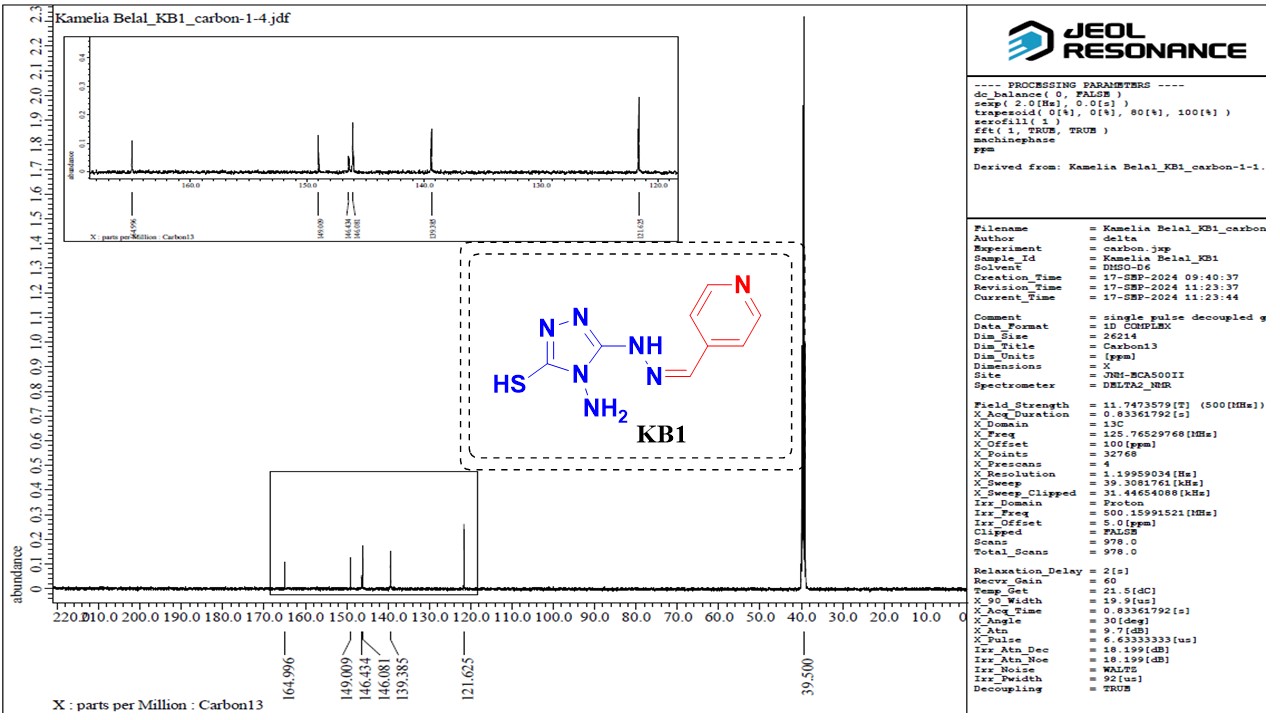
**S1**. ^1^H-NMR spectrum of compound KB1.

**S2**. ^13^C-NMR spectrum of compound KB1.


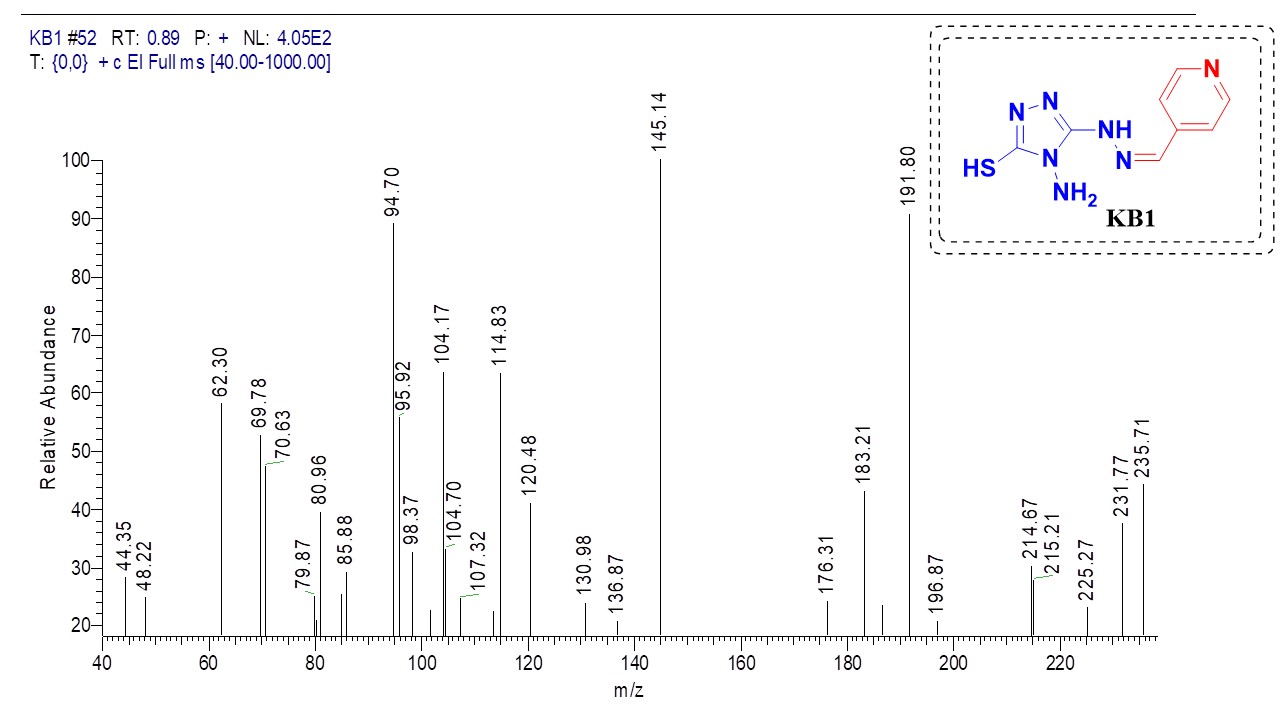


**S3**. Mass spectrum of Compound KB1.


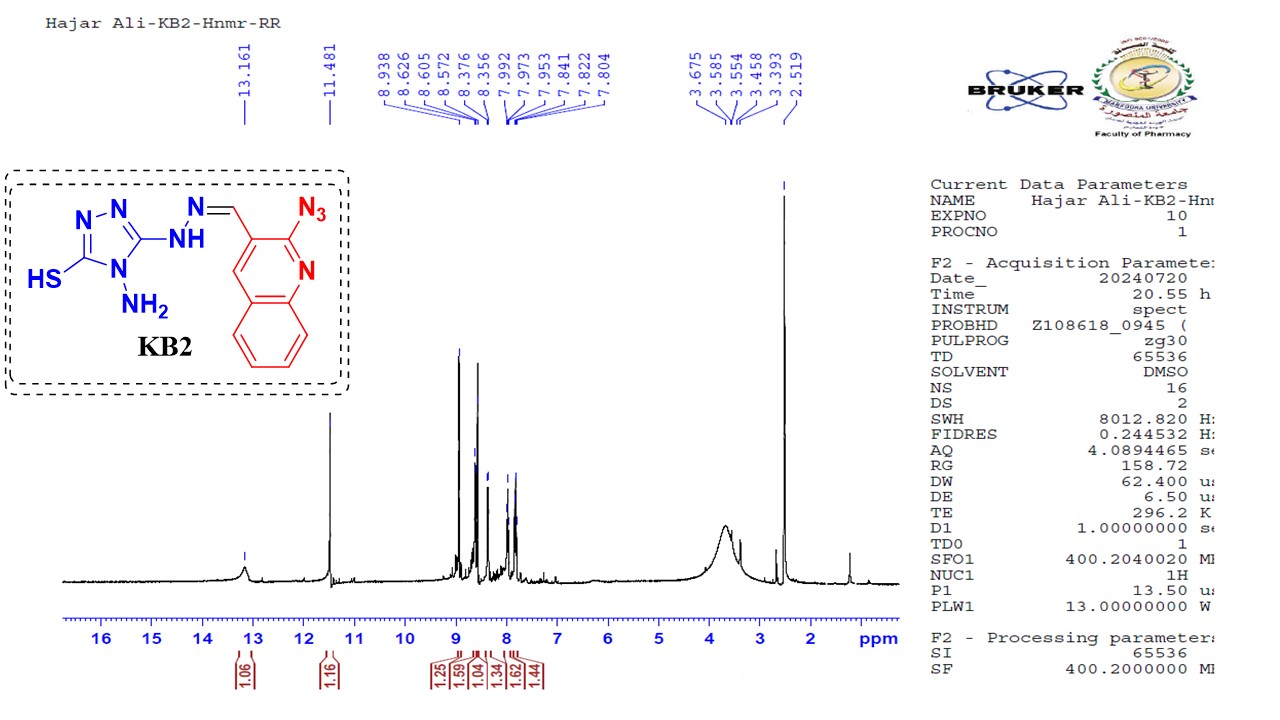


**S4**.^1^H-NMR spectrum of compound KB2.


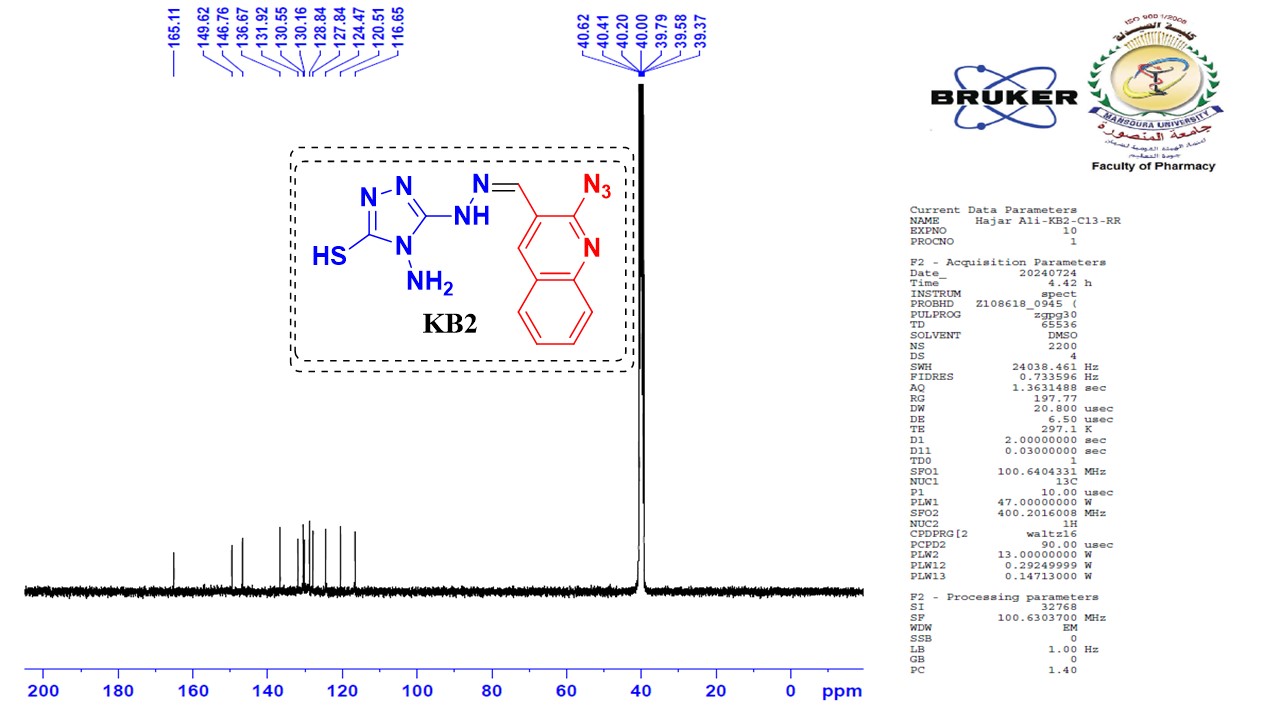
**S5**. ^13^C-NMR spectrum of compound KB2.


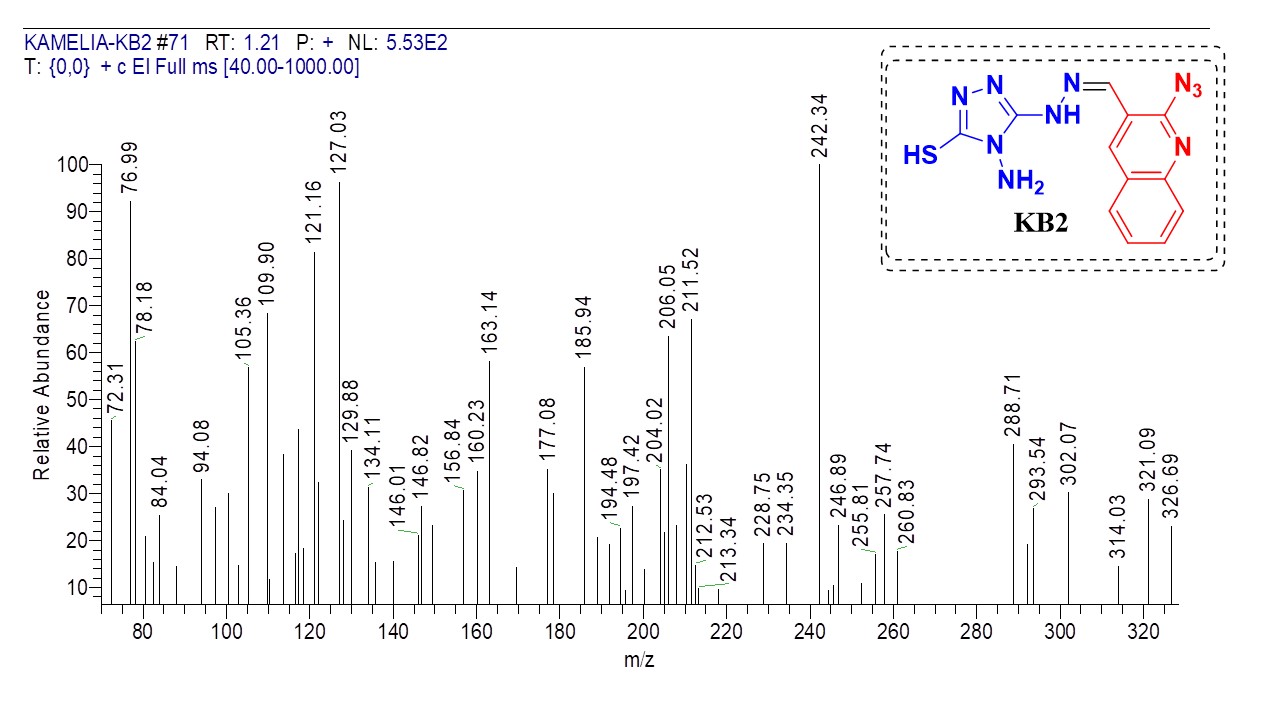


**S6**. Mass spectrum of Compound KB2.

**Synthesis process of the inhibitors**

The reaction conditions of the synthesis of inhibitors KB1 and KB2 were screened using other acidic catalysts, such as (conc. HCl, CF_3_CO_2_H), without any benefits in both yield and reaction time. Furthermore, we found that the alkaline catalyst did not affect the reaction, confirming that a strong acidic medium was an indispensable condition of the protocol methodology. In order to optimize reaction conditions of triazole **1** with pyridine-4-carboxaldehyde (**2**) or 2-azidoquinoline-3-carbaldehyde (**3**), the effect of solvent was conducted involving various polar and non-polar solvents (distilled water, petroleum ether, ethyl acetate, dioxane, Et_2_O, CH_3_CN, AcOH, CHCl_3_, DMF, DMSO, toluene). As the results of the optimization of the reaction conditions showed no product was obtained in the absence of the catalyst, and some of the other mentioned solvents do not dissolve triazole as our starting material (distilled water, petroleum ether, ethyl acetate, dioxane, Et_2_O, CH_3_CN). Whereas other solvents led to various side reactions requiring tedious chromatographic purification. In addition, the potential variability in the process for synthesis of the targeted inhibitors was investigated through both ultrasound and conventional methods, as illustrated in **Table S1**. Upon repeating the experiments with a newly prepared sample of inhibitors, identical results were obtained, confirming the reproducibility of the results.

Table caption

**Table S1** Time and yield comparison between conventional and ultrasound irradiation methods for compounds KB1 and KB2.

| Compound | Ultrasound Method | | Convention Method | |
| --- | --- | --- | --- | --- |
|  | Time (min) | Yield (%) | Time (min) | Yield (%) |
| KB1 | 3 | 92 | 7 | 83 |
| KB2 | 3 | 91 | 9 | 79 |
